# Dynamic stabilization of a mechanical oscillator in the absence of any stable feature

**DOI:** 10.1038/s41467-026-70493-1

**Published:** 2026-03-10

**Authors:** David Xiedeng, Paolo Celli, Maurizio Porfiri

**Affiliations:** 1 Department of Civil Engineering, Stony Brook University, Stony Brook, NY USA; 2 Center for Urban Science and Progress, Tandon School of Engineering, New York University, Brooklyn, NY USA; 3 Department of Biomedical Engineering, Tandon School of Engineering, New York University, Brooklyn, NY USA; 4 Department of Civil, Urban and Environmental Engineering, Tandon School of Engineering, New York University, Brooklyn, NY USA; 5 Department of Mechanical and Aerospace Engineering, Tandon School of Engineering, New York University, Brooklyn, NY USA

**Keywords:** Mechanical engineering, Applied physics

## Abstract

How and why base vibration can stabilize an inverted pendulum has puzzled the scientific community for decades, until the work on dynamic stabilization by Pyotr Kapitza pointed at the alternation between unstable and stable modes as a pathway to stability. We report the discovery of a mechanical oscillator that switches between two unstable modes, has an unstable average, and, yet, can be dynamically stabilized. Our system is governed by a modified Meissner’s model – a one-degree-of-freedom oscillator where both stiffness and damping are modulated through a square wave to switch between positive and negative values. We theoretically prove the existence of compact antiresonance windows and provide experimental evidence through a cantilever beam oscillator subject to magnetic and aerodynamic forcing. The prospect of dynamic stabilization in the absence of any stable feature has vast implications from network dynamical systems, to structural mechanics and robotics.

## Abstract (web summary)

The study demonstrates dynamic stabilization of a mechanical oscillator switching between two unstable modes using time-modulated stiffness and damping. It provides theoretical and experimental evidence of antiresonance windows, enabling stability without any stable mode or average.

## Introduction

Open-loop control – a strategy in which the control action is independent of the system’s output – has been the cornerstone of mechanical engineering design for centuries, from the clepsydras (water clocks) used by ancient civilizations to measure time^1^ to the mechanical automata that paved the way to modern robotics^2^. Mastery of mechanics principles enabled engineers to design machines that would perform desired tasks without the need to self-correct via feedback mechanisms. For example, in 1478, Leonardo da Vinci designed what is likely the first example of a robot in western civilization, as reported one century later by artist and writer Gian Paolo Lomazzo^2^: “once in front of Francis I, King of France, [Leonardo] caused a lion, constructed with marvelous artifice, to walk from its place in a room and then stop, opening its breast which was full of lilies and different flowers.” The lion was the exquisite integration of several elements of engineering knowledge in the Renaissance, particularly the design of cam systems that would allow the robot to perform desired movements.

**Fig. 1:**
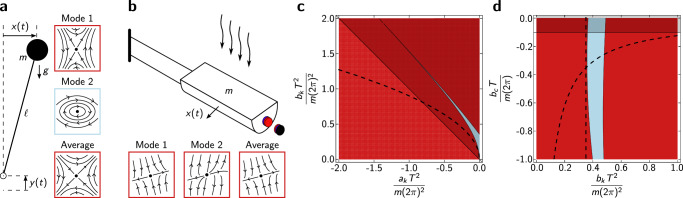
Dynamic stabilization of Kapitza’s pendulum and of the proposed mechanical oscillator. **a** Sketch of a Kapitza’s pendulum of length *ℓ*, where *x* is the lateral displacement of the mass *m* from the vertical line, *g* is gravity, and *y* is the base excitation. For a square-wave modulation, the inverted pendulum is stabilized by switching between a saddle and a center, with the average mode being a saddle. **b** Proposed mechanical oscillator, consisting of a mass *m* at the tip of a cantilever beam. The tip mass is subjected to aerodynamic loading and magnetic forces, which switch in time to modulate both stiffness and damping, as in our modified Meissner’s model. The oscillator is stabilized by switching between a saddle and an unstable focus (or an unstable node), with the average mode being a saddle. **c**, **d** Stability maps for the classical and modified Meissner’s models, for a duty cycle $\delta=\frac{1}{2}$, illustrating the formation of parameter regions (light blue) where the system is stable; red regions identify time-modulations leading to unstable response. In (**c**), the transparent triangle below the solid line is the region in which both modes are unstable. The dashed curve is the estimate of the stability boundary through the theory of motion in a rapidly evolving field, ${b}_{k} > \frac{\sqrt{2| {a}_{k}| m}}{P}\frac{2\pi }{T}$, highlighting that stability requires the stiffness modulation *b*_*k*_ to dominate the average stiffness *a*_*k*_ and create a center to fall back on during switching. In (**d**), we set $\frac{{a}_{k}{T}^{2}}{m{(2\pi )}^{2}}=-\frac{1}{10}$ and $\frac{{a}_{c}T}{m(2\pi )}=\frac{1}{10}$. The transparent rectangle below the solid line is the region in which both modes are unstable. The dashed curves are estimates of the stability boundaries through the theory of motion in a rapidly evolving field, ${b}_{k} > \frac{\sqrt{2| {a}_{k}| m}}{P}\frac{2\pi }{T}$ and ${b}_{c} > -\frac{2{a}_{c}m}{{P}^{2}{b}_{k}}\frac{{(2\pi )}^{2}}{{T}^{2}}$.

Compared to feedback control systems^3^, open-loop control systems are easier to design, more economic to realize, and faster to operate. The most common critique to open-loop control systems is that they are sensitive to external disturbances, whereby their response may be dramatically affected by changing conditions and exogenous factors. Yet, this alleged fragility is not common to all open-loop systems. Named after the Russian Nobel Prize laureate physicist Pyotr Kapitza, Kapitza’s pendulum^4^ is an inverted pendulum that can be robustly stabilized in open-loop, see Fig. 1a and Methods. For a moderate base oscillation amplitude, there is a critical oscillation frequency above which the inverted configuration is stable. The dynamic stabilization of the inverted configuration is related to the fast vibration of the base that modulates the stiffness of the system, creating a well in the effective potential on a slow scale dynamics^5^. The curvature of the well, that is, the effective stiffness, is proportional to the square of the product between the amplitude and the frequency of the base excitation. In this vein, for sufficiently fast modulations, the well will dominate the effective potential, leading to stability. Such a slow-fast dichotomy can be described through Meissner’s^6^ or Mathieu’s equation^7^. Both equations are special forms of the Hill’s equation^8^ – the former implementing square-wave and the latter harmonic modulation of the stiffness term^9–11^. The principles underpinning Kapitza’s pendulum have been extensively studied in physics, where it represents the basis for devices like cyclotrons^12^ and ion traps^13^ and in engineering, where it informs the design of flying robots^14^, levitating fluids^15^, active electric circuits^16^, and qubits^17^.

The fast base excitation required to stabilize Kapitza’s pendulum is practically equivalent to a restoring force that counterbalances gravity for a fraction of the oscillation cycle. Should one view the dynamics simply as the alternation between two modes, akin to the Meissner’s model, they would argue that dynamic stabilization of Kapitza’s pendulum leverages the switching between an unstable (saddle) and a neutrally stable mode (center), see ”Methods”. What if the positive effect of the center is absent and the system can only alternate between unstable modes? Is dynamic stabilization still feasible? We provide a positive answer to this question through the introduction of negative damping in the dynamic stabilization framework. Negative damping is unusual, but not unheard of in engineering, where it is used to model the squealing of railway wheels due to self-excited vibrations^18^, structural vibrations due to buffeting by fluid eddies or wake turbulence from upstream bodies^19^, and pedestrian-induced instability of bridges^20^. By allowing for the possibility to simultaneously modulate the stiffness and damping of a mechanical system, we open the door to a form of dynamic stabilization in which the system is stable only for compact windows of excitation frequency–despite all of its modes and its time-average being unstable. Different from Kapitza’s pendulum, there is no lower bound for the excitation frequency above which one can advocate for the stabilization of the slow dynamics. Dynamic stabilization arises due to the alternation between a saddle and an unstable focus or an unstable node, for excitation frequencies within narrow “antiresonance” windows that favor decaying dynamics along the principal stable direction of the saddle.

We demonstrate this form of dynamic stabilization both theoretically and experimentally. We propose a variation of Meissner’s model, in which both the damping and stiffness are periodically modulated in time through a square wave. The intuition behind the use of a switched damping is to induce a qualitative asymmetry between the two modes, which may facilitate steering the dynamics of the system to the origin in the case of unstable modes. With this model, we predict the emergence of antiresonance windows, combining unstable modes of different nature (a saddle and either an unstable focus or an unstable node). Our theoretical findings are confirmed by experiments on a cantilever beam oscillator, in which negative stiffness is generated via magnetic-force-induced bistable mechanics and negative damping via flow-induced galloping instability, see Fig. 1b. Our mechanical oscillator constitutes a critical step beyond Kapitza’s pendulum, illustrating dynamic stabilization in the absence of a stable feature, whether it is a mode or the average.

## Results

### Theory

In the study of one-degree-of-freedom (1-DOF) linear oscillators, one typically makes the assumption that all the parameters are positive and time invariant. In this vein, one examines a 1-DOF oscillator of the form $m\ddot{x}(t)+c\dot{x}(t)+kx(t)=0$, where *x*(*t*) is the state of the system as a function of time, *m* is the mass, *c* is the damping, and *k* is the stiffness. For any parameter choice, the system is (asymptotically) stable (both the eigenvalues of the second-order characteristic equation have negative real part), so that any initial condition in the phase space approaches the origin of the phase plane^21^.

If at least one parameter between *k* and *c* is negative, the oscillator becomes unstable (one of the eigenvalues has positive real part). If *k* < 0, then the origin of the phase plane is a saddle (real eigenvalues: one positive and one negative). If *k* > 0 and *c* < 0, then there are two possibilities depending on the value of the discriminant $\Delta :=\frac{{c}^{2}}{{m}^{2}}-4\frac{k}{m}$. If Δ < 0, the origin is an unstable focus (complex conjugate eigenvalues with positive real part). If Δ > 0, the origin is an unstable node (real eigenvalues, both positive).

The simplest form of time-modulation for a 1-DOF oscillator is through switching of model parameters, as Meissner originally proposed over a century ago to study the oscillations of side rods of locomotives^6^. Meissner’s model consists of an undamped oscillator of constant mass, whose stiffness switches periodically between two values. Through Floquet theory, one can unveil a rich dependence of the system stability on the model parameters (switching period and stiffness values), including parametric resonance for select values of the switching period even when switching between stable modes (positive stiffness)^11^. The addition of a constant positive damping to Meissner’s equation modifies the stability boundaries, destabilizing some regions akin to the Ziegler effect observed for damped gyroscopic systems^22^.

We consider an extension of the classical Meissner’s model in which both the stiffness and damping vary in time. We allow for both stiffness and damping to attain negative values to control the stability of the origin. In particular, considering negative damping opens the door to switching between three different types of instability (saddle, unstable focus, and unstable node); a positive damping would preclude the emergence of either an unstable focus or an unstable node. Hence, we study the following open-loop switched dynamics:

1 $$m\ddot{x}(t)+\left({a}_{c}+p(t){b}_{c}\right)\dot{x}(t)+\left({a}_{k}+p(t){b}_{k}\right)x(t)=0.$$

Here, *a*_*k*_ and *a*_*c*_ are the average stiffness and damping, *b*_*k*_ and *b*_*c*_ measure the extent to which the two parameters of the two modes differ, and *p*(*t*) is a periodic, zero-mean square wave time-modulation,

2 $$p(t)=\left\{\begin{array}{l}-2\delta \,\,{{{\rm{if}}}}\,\,0 < t < (1-\delta )T\\ 2(1-\delta )\,\,{{{\rm{if}}}}\,\,(1-\delta )T < t < T\end{array}\right.,$$

with *T* being the switching period and *δ* the duty cycle, between 0 and $\frac{1}{2}$. With this notation, the damping and stiffness of the first mode are *c*_1_ = *a*_*c*_ − 2*δb*_*c*_ and *k*_1_ = *a*_*k*_ − 2*δb*_*k*_; those of the second mode are *c*_2_ = *a*_*c*_ + 2(1 − *δ*)*b*_*c*_ and *k*_2_ = *a*_*k*_ + 2(1 − *δ*)*b*_*k*_.

Some intuition about the stability of the modified Meissner’s model in (1) can be gathered by applying the theory of motion in a rapidly evolving field of Landau and Lifshitz^5^, see Methods. First, we approximate the time-modulation as a single harmonic of radian frequency $\omega=\frac{2\pi }{T}$ and amplitude $P=\frac{4\sqrt{{\sin }^{2}(\pi \delta )}}{\pi }$. We assume the time modulation to be fast with respect to the characteristic time scale of the average system, which is dictated by the interplay between stiffness and damping. Specifically, one should compute the smallest between ${\tau }_{0,1}=\sqrt{\frac{m}{| {a}_{k}| }}$ and ${\tau }_{0,2}=\frac{m}{| {a}_{c}| }$ to identify the characteristic time scale of the system, *τ*_0_. Within a first approximation, when *τ*_0,1_ < *τ*_0,2_, stiffness dominates damping, and, vice versa, when *τ*_0,1_ > *τ*_0,2_, damping dominates stiffness. We decompose the dynamics into a fast, small, and zero-mean oscillation, *ξ*(*t*), and a slow, smooth motion, *X*(*t*). Carrying the steps, we establish the following equation for the slow dynamics, see Methods:

3 $$\ddot{X}(t)+\frac{{a}_{c}}{m}\left(1+\frac{{P}^{2}{b}_{k}{b}_{c}}{2{a}_{c}m{\omega }^{2}}\right)\dot{X}(t)+\frac{{a}_{k}}{m}\left(1+\frac{{P}^{2}{b}_{k}^{2}}{2{a}_{k}m{\omega }^{2}}\right)X(t)=0.$$

Like Kapitza’s pendulum, the stiffness modulation always offers a stabilizing effect, raising the average stiffness by $\frac{{P}^{2}{b}_{k}^{2}}{2{\omega }^{2}}$. The damping modulation, instead, may be beneficial or detrimental to stability, depending on whether it is in phase (*b*_*k*_ and *b*_*c*_ have the same sign) or out of phase (*b*_*k*_ and *b*_*c*_ have opposite sign) with the stiffness modulation, respectively. The latter case offers an interesting scenario for stability: when the average system is a saddle (*a*_*k*_ < 0) and *a*_*c*_ > 0, time-modulation can be employed to stabilize the switched system alternating between unstable modes. Specifically, assuming *b*_*k*_ > 0 and *b*_*c*_ < 0, stability is guaranteed by ${b}_{k} > \frac{\sqrt{2| {a}_{k}| m}}{P}\frac{2\pi }{T}$ and ${b}_{c} > -\frac{2{a}_{c}m}{{P}^{2}{b}_{k}}\frac{{(2\pi )}^{2}}{{T}^{2}}$; we can find combinations of the parameters (including *T*, *b*_*k*_, and *b*_*c*_) to satisfy these two conditions in a manner that ensures *a*_*c*_ + 2(1−δ) *b*_*c*_ < 0 so that the system alternates between two unstable modes (one with negative stiffness, *a*_*k*_ − 2*δ b*_*k*_ < 0, and one with negative damping, *a*_*c*_ + 2(1−*δ*) *b*_*c*_ < 0). In this vein, stability relies on fine tuning of Kapitza’s effect to create a positive average stiffness, without triggering a negative average damping.

Averaging methods are helpful in building intuition about system dynamics, but their predictions should always be verified against rigorous stability theory. Borrowing words from the analysis of Wickramasinghe and Berg^23^ on harmonically excited Kapitza’s pendulum: “nonlinear methods, which typically rely on asymptotic stability theorems, address only a small part of the stability map.” To assess the stability of the modified Meissner’s model in (1) and (2), we examine the eigenvalues of the monodromy matrix *Φ*(*T*)^21^, defined as $\Phi (T)=\exp (\delta T{A}_{2})\exp ((1-\delta )T{A}_{1})$, with $${A}_{1}=\left[\begin{array}{cc}0 & 1\\ -\frac{{k}_{1}}{m} & -\frac{{c}_{1}}{m}\end{array}\right]$$ and $${A}_{2}=\left[\begin{array}{cc}0 & 1\\ -\frac{{k}_{2}}{m} & -\frac{{c}_{2}}{m}\end{array}\right]$$ being the two-by-two state matrices for the first and second mode, respectively.

For two-by-two switched systems with real coefficients, one can establish general closed-form conditions for stability that extend previous results on the Meissner’s model^11^. The necessary and sufficient condition for the (asymptotic) stability of the switched system is that the eigenvalues of *Φ*(*T*) (*ρ*_1_ and *ρ*_2_, typically called Floquet multipliers) are inside the unit circle^21^. The Floquet multipliers can be written in terms of the two invariants (determinant, $\det$, and trace, ${{{\rm{tr}}}}$), as ${\rho }_{1}{\rho }_{2}=\det \Phi (T)$ and ${\rho }_{1}+{\rho }_{2}={{{\rm{tr}}}}\Phi (T)$. Acknowledging that *ρ*_1_ and *ρ*_2_ form a complex conjugate pair or are both real numbers, we can prove that ∣*ρ*_1,2_∣ < 1 is equivalent to (see Methods for a proof)

4a $$| \det \Phi (T) < 1$$

4b $$| {{{\rm{tr}}}}\Phi (T)| < 1+\det \Phi (T).$$

For the modified Meissner’s model in (1) and (2), the determinant and trace of the monodromy matrix take simple, compact forms that are amenable to analytical insight,

5a $$\det \Phi (T)=\exp \left(\frac{-{a}_{c}T}{m}\right),$$

5b $${{{\rm{tr}}}}\Phi (T)=	 \exp \left(-\frac{{a}_{c}T}{2m}\right)\times \left[(1+\theta )\cosh \left(\frac{(1-\delta )\sqrt{{\Delta }_{1}}+\delta \sqrt{{\Delta }_{2}}}{2}T\right) \right. \\ 	 \left.+\right.\left.(1-\theta )\cosh \left(\frac{(1-\delta )\sqrt{{\Delta }_{1}}-\delta \sqrt{{\Delta }_{2}}}{2}T\right)\right],$$

where ${\Delta }_{1,2}:=\frac{{c}_{1,2}^{2}}{{m}^{2}}-4\frac{{k}_{1,2}}{m}$ and $\theta :=\frac{\frac{{c}_{1}{c}_{2}}{{m}^{2}}-2\frac{({k}_{1}+{k}_{2})}{m}}{\sqrt{{\Delta }_{1}{\Delta }_{2}}}$. Regions of the parameter space in which the switched system is stable – often called Arnold tongues^24^ – are obtained by fulfilling conditions in (4) using the expressions given in (5).

In the case where the average system is a saddle, the classical Meissner’s model (*a*_*c*_ = *b*_*c*_ = 0) describes a Kapitza’s pendulum (see Methods). In Fig. 1c, we illustrate (marginal) stability regions for this model, which can be mapped into stability regions for Kapitza’s pendulum by setting ${a}_{k}=-\frac{mg}{\ell }$ and $p(t){b}_{k}=\frac{m}{\ell }\ddot{y}(t)$. We highlight two different regions in the stiffness parameter space: both modes are unstable (both saddles) in the bottom-left triangle; one mode is neutrally stable and the other is unstable (a center and a saddle) in the shaded region. Stable regions for the switched system do not exist if both modes are unstable: at least one of the modes must be stable. In agreement with previous work^23,25^, we find that the theory of motion in a rapidly evolving field captures the lower threshold of stiffness modulation to attain a stable response, but it does not anticipate the emergence of Arnold tongues.

Considering the presence of negative damping, one can obtain dynamic stabilization without any of the modes being stable. In Fig. 1d, we present stability regions obtained by including negative damping in the modified Meissner’s model. By doing so, one obtains large stability regions where the switched system is stable, despite both its modes being unstable. The theory of motion in a rapidly evolving field^5^ is successful in capturing the lower threshold for the stiffness modulation and in pointing to the destabilizing role of out-of-phase stiffness and damping modulations; yet, it could miss salient features of the stability landscape (see Supplementary Note 1 and Supplementary Fig. 1 for a comparison).

**Fig. 2:**
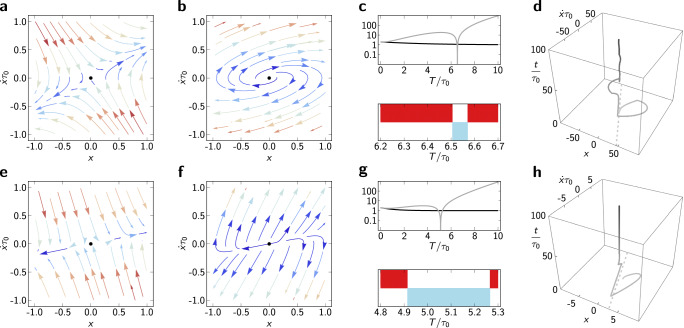
Dynamic stabilization of two unstable modes with an unstable average. **a**−**d** Saddle and unstable focus ($m=10\,{{{\rm{kg}}}},{a}_{k}=-1\,{{{\rm{N}}}}\,{{{{\rm{m}}}}}^{-1},{b}_{k}=\frac{3}{2}\,{{{\rm{N}}}}\,{{{{\rm{m}}}}}^{-1},{a}_{c}=1\,{{{\rm{N}}}}\,{{{\rm{s}}}}\,{{{{\rm{m}}}}}^{-1},{b}_{c}=-2\,{{{\rm{N}}}}\,{{{\rm{s}}}}\,{{{{\rm{m}}}}}^{-1}$, and $\delta=\frac{1}{2}$); **e**−**h** saddle and unstable node (*m* = 10 kg, *a*_*k*_ = − 1 N m^−1^, *b*_*k*_ = 2 N m^−1^, *a*_*c*_ = 4 N s m^−1^, *b*_*c*_ = − 10 N s m^−1^, and $\delta=\frac{2}{5}$). Streamlines of the (**a**, **e**) first and **b**, **f** second modes for the two considered instances: **a** saddle and **b** unstable focus, and **e** saddle and **f** unstable node. **c**, **g** Illustration of the transcendental equation in condition (4b) that defines the emergence of antiresonance for the switched system (gray: $| {{{\rm{tr}}}}\Phi (T)|$ and black: $1+\det \Phi (T)$), along with a zoomed-in view of the stability map (red: unstable; light blue: stable). Illustration of the time-response in the phase space for: **d** the oscillator switching between a saddle and an unstable focus for *T* = 6.55*τ*_0_, with *τ*_0_ = 3.16 s; and **h** the one switching between a saddle and an unstable node for *T* = 5.09*τ*_0_, with *τ*_0_ = 2.5 s. In both cases, initial conditions are *x*(0) = 1 and $\dot{x}(0)=0$ (units for *x* are arbitrary given that the problem is linear), and the dashed red line indicates the stable principal direction of the saddle.

The phenomenon we unveil is not based on the fast switching limit; in fact, fast switching begets the stability landscape of the average system (a saddle)^26^. Rather, we report the existence of compact windows of switching periods $[{T}_{\min },{T}_{\max }]$ of antiresonance, in which the switched system is stable even though both modes and the average are unstable. Condition (4a) requires that *a*_*c*_>0, but does not place any constraint on *a*_*k*_. The average system can be a saddle (*a*_*k*_ < 0) and, yet, the switched system can be stable at intermediate switching periods. If mode 1 is a saddle (with positive damping) and mode 2 is an unstable focus, then Δ_1_ > 0 and Δ_2_ < 0. In this case, the trace of the monodromy in (5b) can switch sign and the emergence of antiresonance can be detected by monitoring such a sign change. In Fig. 2a–d, we demonstrate the occurrence of antiresonance over a relatively broad range of switching periods. Importantly, the trajectory of the switched system does not approach the origin monotonically, whereby we register dramatic oscillations during the decay towards the origin. The stable dynamics of the switched system consist of diverging clockwise rotations in the phase space when the unstable focus is active, followed by converging dynamics along the stable principal direction of the saddle. A similar scenario is discovered when mode 1 is a saddle (with positive damping) and mode 2 is an unstable node, Δ_2_ > 0, see Fig. 2e–h.

One’s intuition would suggest that stability should benefit from switching between two saddles with different principal directions, alternating converging dynamics towards the origin. However, this is not the case. If both modes are saddles–with an average positive damping–we do not find a switching period for which the switched system is stable. This finding is supported by the analysis of the functions in (5a) and (5b), see Methods, and it can be explained with the theory of motion in a rapidly evolving field by examining the effective stiffness in (3). Just like the base excitation in Kapitza’s pendulum has to ensure the existence of a stable mode, see Methods, so the stiffness modulation in the modified Meissner’s model should be sufficiently strong to ensure the existence of an unstable focus or an unstable node. An even more intuitive explanation for this counterintuitive finding may be put forward by examining the phase portraits. A saddle has two principal directions: an unstable one crossing the first and third quadrants and a stable one crossing the second and fourth quadrants. Changing the negative stiffness can vary the slope of the principal directions, but never enough to make them cross the axes. As such, there are no switching patterns that allow a trajectory to rotate towards the origin when alternating between two saddles. If the trajectory is in the first or third quadrant, switching will not modify the approaching motion toward the unstable principal direction. Likewise, if the trajectory is in the second or fourth quadrant, switching will not rotate the trajectory toward the stable principal direction. If one of the modes is changed to an unstable node or to an unstable focus, we allow the possibility to rotate the trajectory towards the origin. Such a rotation, combined with the presence of a stable principal direction of the saddle, opens the door to stabilizing intermediate switching.

### Design of the mechanical oscillator

**Fig. 3:**
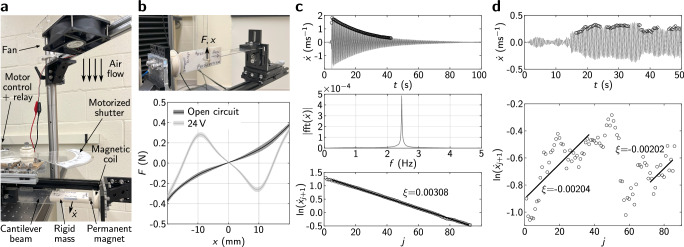
Experimental setup and parameter estimation. **a** 1-DOF oscillator consisting of a rigid mass connected to a cantilever beam and subjected to aerodynamic loading (induced by the air flow from the top fan) and magnetic forces (due to the repulsive interaction between the external magnetic coil and permanent magnets connected to the mass). **b** Experimental setup for stiffness measurement, along with average and standard deviation (shaded areas) of the force-displacement curves with the magnetic coil off (open circuit) and on (24 V). **c** Free response of the system with positive stiffness and positive damping, including a time-history (gray curve, with markers identifying peaks), a frequency response, and a plot of the natural logarithm of the values of the peaks of the response against the peak number (markers), which is used to estimate damping via the logarithmic decrement procedure (fitting the straight black line onto the peak data). **d** Response of the system subjected to the fan-induced air flow, together with the logarithmic decrement plot used to estimate the negative damping.

Providing experimental evidence of dynamic stabilization in the absence of stable modes requires the design of a mechanical system in which both stiffness and damping can be dynamically switched between positive and negative values. We realize a 1-DOF oscillator in the form of a rigid mass, attached to the tip of a cantilever beam and subjected to both aerodynamic and magnetic loading. The tip mass has a half-cylindrical shape and its rectangular face is aligned with the beam’s weak axis, as shown in Fig. 3a and, more in detail, in Supplementary Fig. 2. For small deformations, the rotation and axial displacement of the beam can be neglected, leaving the lateral displacement of the tip mass as the only DOF. The beam motion is recorded via a single-point laser vibrometer, which acquires the lateral velocity $\dot{x}$ as a function of time.

Negative damping is induced via galloping instability, by subjecting the tip mass to a fan-generated air flow normal to its rectangular face^27^. Vortex shedding is known to manifest when laminar flow interacts with bluff bodies of specific cross sections^28^. The flow effectively applies a force onto the structure^29^ that is in phase with its velocity and adds energy to the system. Conversely, negative stiffness is introduced by adding a permanent magnet to the extremity of the tip mass, and by fixing a magnetic coil at a set distance from the permanent magnet–the tip mass, including the permanent magnets is *m* = 0.049 kg. Turning the coil on induces a repulsive force onto the permanent magnet, so that the system becomes bistable^30^ with a negative effective stiffness around its zero-displacement position^31,32^.

Our system is programmed in open-loop to switch between a saddle (mode 1: *k*_1_ < 0 and *c*_1_ > 0) and another unstable focus or node (*k*_2_ > 0 and *c*_2_ < 0) for $\delta=\frac{1 }{2}$. The stiffness can be switched by controlling the current through the magnetic coil with a square waveform at a desired period. This is achieved with an Arduino-controlled relay circuit, as discussed in Methods and as shown in Supplementary Fig. 3. Switching the damping values is not as simple, as the fan has a start-up transience that is much longer than the switching periods of interest. To alleviate this issue, we design a servomotor-controlled shutter–a flat disk shape in which two opposite quarters of it have been removed. As it rotates, the shutter periodically blocks the air flow produced by the fan. The shutter and magnetic coil are then synchronized via a procedure described in Methods and Supplementary Fig. 4.

Model parameters are identified through a series of sequential steps. To measure the positive and negative stiffness, we rotate the support without moving the beam, as shown in Fig. 3b. We utilize a pre-made hole in the tip mass to anchor it to a custom gripper (shown more in detail in Supplementary Fig. 5), which we connect to a Universal Testing System (UTS) to measure the force-displacement curve of our mechanical system for various voltages supplied to the magnetic coil. We perform three experimental repetitions for the identification of the positive and negative stiffness. Open-circuiting the magnetic coil, we identify the positive stiffness *k*_2_ = 10.74 ± 2.76 N m^−1^, while applying 24 V across the coil, we determine the negative stiffness *k*_1_ = − 23.68 ± 3.54 N m^−1^.

Whether positive or negative, damping is identified by open-circuiting the magnetic coil and studying the logarithmic decay or growth of the tip mass velocity in time. Specifically, we track the peaks of the time-trace and estimate the damping ratio *ξ*_1,2_ using the logarithmic decrement procedure. The latter consists in fitting the slope of the curve obtained by plotting the logarithm of the response peaks versus the peak number, as also shown in Fig. 3c. From the damping ratio, one determines the damping as ${c}_{1,2}=2{\xi }_{1,2}\sqrt{{k}_{2}m}$; note the consistent use of the positive stiffness given that the magnet is inactive. The positive damping ratio is estimated by covering the beam with the shutter and subjecting the tip mass to a small initial displacement; the time-evolution of the beam is shown in Fig. 3c, together with its single-peak frequency response. By repeating the experiment three times (additional data is shown in Supplementary Fig. 6a–c), we estimate the positive damping ratio *ξ*_1_ = 0.0031 ± 0.0002. The negative damping is instead measured by opening the shutter, so that the air flow excites the structure. The time-evolution of the beam in this scenario is shown in Fig. 3d. In comparison to the positive damping measurements, where the envelope of the time-trace decays monotonically, here the envelope of the time-trace fluctuates over time. To locate regions where there is an overall growth in response, we apply a kernel-based smoothing algorithm to the data–see Methods for details–and use the positive slopes of the smoothened data to extract damping ratios. By repeating the experiment three times (additional data is shown in Supplementary Fig. 6d–f), we extract a negative damping ratio *ξ*_2_ = − 0.0024 ± 0.0010, which causes mode 2 to be an unstable focus, that is, ${\Delta }_{2}=4({\xi }_{2}^{2}-1)\frac{{k}_{2}}{m} < 0$.

### Evidence of dynamic stabilization

Prior to investigating dynamic stabilization, we verify that both modes are unstable. Time-traces, like those in Fig. 3d and Supplementary Fig. 6d–f, show that the response of the system in its second mode oscillates but keeps on growing–a sign of instability. Evidence for instability of the first mode is instead provided in Supplementary Fig. 7: when 24 V are supplied to the coil, the system leaves its rest position and moves to one of the stable equilibria of the now-bistable energy landscape (estimated from the 24 V curve in Fig. 3b to be located at *x* = ± 15 mm, where the curve crosses the zero-force axis with a positive slope).

**Fig. 4:**
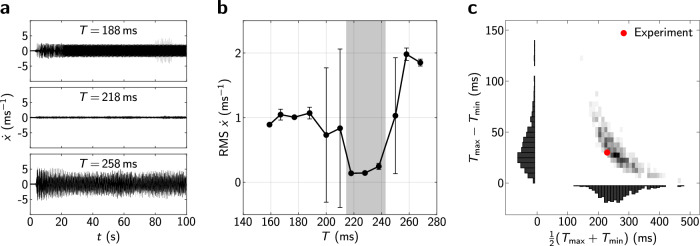
Experimental evidence of stabilization of our mechanical oscillator and comparison to theory. **a** Time-histories for three representative switching periods; each plot features time-traces from three experiments, in different shades of gray. **b** Plot of the average and standard deviation of the RMS of the velocity time-traces, against the switching period; the shaded area indicates the stable region where the system oscillates around its rest position. **c** Statistical distribution for the center and range of the stability region, obtained using the modified Meissner’s model by varying the system’s parameter within the experimentally measured bounds. The colormap represents the joint probability mass function, with darker colors indicating higher values; the red dot indicates experimental findings, extracted from (**b**).

We test our system for a range of switching periods between 159 ms and 268 ms. For each period, we initially turn the fan and the magnetic coil’s power supply on, cover the beam with the shutter, place the beam in its rest position, and, finally, we start the test by turning on the switching circuit. Each experiment is repeated three times. We record velocity time-traces over 100 s, as shown in Fig. 4a; we then compute the root mean square (RMS) of each time-trace and the mean and standard deviation of the RMS for each switching period, as summarized in Fig. 4b. We register a strong dip in the RMS for switching periods between 218 and 238 ms–providing evidence in favor of dynamic stabilization in a compact antiresonance window. The time-traces in Fig. 4a are selected to represent responses before, within, and after the antiresonance window, thereby highlighting the difference between stable and unstable regimes. Inspection of the recorded videos of the response provided as Supplementary Videos and described in Supplementary Note 2 show that, within the antiresonance window, the beam remains in the neighborhood of its rest position. Outside of that window, the beam moves towards the stable equilibria of the negative stiffness configuration. For switching close to the transition between stable and unstable regimes (200, 210, and 250 ms), we observe a large variation in the RMS among trials–whereby one of the acquisition shows a dramatically unstable behavior (see Supplementary Fig. 8 and Supplementary Note 3). This is not surprising, given the experimental uncertainty in the modulation signal and especially the fact that the shutter unlikely provides a perfect square wave modulation for the damping.

Comparing our experimental findings on the antiresonance window (${T}_{\min }=214$ ms and ${T}_{\max }=244$ ms) to theoretical predictions requires accounting for the experimental uncertainty accrued in the identification of the system’s parameters. To do so, in our model, we independently vary parameters *k*_1_, *k*_2_, *c*_1_, and *c*_2_ within their experimental uncertainty ranges, following a normal distribution, while keeping the mass constant and excluding combinations that would violate the necessary conditions for the stability of the modified Meissner’s model (*a*_*c*_ < 0 and 2(1−*δ*) *b*_*c*_ > − *a*_*c*_). For each combination of parameters, we theoretically evaluate the bounds of the antiresonance region, and we repeat this process 1000 times. We obtain statistical distributions of the center $\frac{1}{2}({T}_{\min }+{T}_{\max })$ and range $({T}_{\max }-{T}_{\min })$ of the antiresonance window, which we plot independently as histograms, and against each other as a colormap in Fig. 4c. This analysis indicates that the most frequent values of the center and range predicted by the theory (the darker regions of the map) are approximately 254 ms and 28 ms, respectively, in good agreement with experimental findings (center: 229 ms and range: 30 ms).

## Discussion

Antiresonance is a key phenomenon in dynamical systems, ubiquitous in physics and engineering. For example, the notion of antiresonance is used to characterize complex integrated quantum circuits^33^, identify squeezing effects in mechanical resonators^34^, and design optical^35^ and acoustic^36^ waveguides with superior mode confinement and selectivity. Typically, antiresonance is associated with destructive interference between multiple components of the system; for instance, the very interaction of a vibration absorber with the host structure is the cause of antiresonant behavior^37^ (that is, reduced vibration of the host structure at select frequencies). However, destructive interference is not the only pathway to create antiresonance in dynamical systems. In the context of the suppression of self-excited vibrations, in the late 1970s, Tondl discovered the stabilizing effect of parametric excitation^38^. The time-modulation of some of the properties of the system induces an energy flow between different modes of the system, through which it is possible to reduce or even suppress self-excited dynamics^39–41^. All the above-mentioned studies documented the onset of antiresonant dynamics in stable systems: whether and how unstable systems can display stable antiresonance are open questions.

In this study, we demonstrate both theoretically and experimentally the emergence of antiresonant behavior in a mechanical oscillator switching between two unstable modes in open loop. Akin to the Kapitza’s pendulum, the oscillator has an unstable average; in contrast to Kapitza’s pendulum, it has no stable mode to fall back on during the time-modulation. Without the need of any feedback system, we demonstrate the feasibility of stabilizing the response of the oscillator through time modulation of both the stiffness and damping in compact antiresonance windows. Dynamic stabilization emerges due to the combined effect of two unstable dynamics: a saddle and either an unstable focus or an unstable node. Saddle dynamics corresponds to a negative stiffness, which we realize via magnetic-force-induced bistable mechanics. The other form of unstable dynamics (unstable focus or node) corresponds to a negative damping, which we create via flow-induced galloping instability.

The theory of motion in a rapidly evolving field offers more insight into the phenomenon. In addition to an increase in the effective stiffness for the slow dynamics due to stiffness modulation, the theory predicts a surprising synergy of stiffness and damping modulation that controls the effective damping. In-phase modulation of damping and stiffness has a stabilizing effect by increasing the effective damping. On the contrary, out-of phase modulation reduces the effective damping, potentially triggering a form instability that has never been documented in the case of dynamic stabilization. Such a destabilizing effect leads to compact antiresonance windows, whereby increasing the excitation frequency above a threshold would lead to an overall negative damping. Combining stiffness increase and damping reduction, one may engineer a stable switched system that alternates between two unstable modes with an unstable average.

To our knowledge, this class of dynamic stabilization has never been documented in the literature. There is extant literature on the fast switching limit of dynamical systems with a stable average^26^, spanning from power converters^42^ to network dynamical systems^43^. Switching between unstable dynamical systems with an unstable average is an uncharted area of research. The closest instance is a modified Stuart-Landau oscillator with a hybrid stable focus-saddle^44^. Due to a lack of rotational invariance, such an oscillator combines features of a stable focus and a saddle, whereby streamlines first approach the origin (like a stable focus) and then escape to infinity along the principal unstable direction (like a saddle). By combining modes that have orthogonal principal unstable directions, it is possible to create antiresonant patterns in which trajectories are attracted toward the origin during the rotation, without reaching the unstable principal directions. The modified Stuart-Landau oscillator of ref. ^44^ is radically different from our mechanical oscillator, being nonlinear and reliant on two hybrid modes of the same class. Through our mechanical oscillator, we demonstrate that antiresonance can appear even in simple and ubiquitous linear systems by combining unstable modes of different nature (saddle with unstable focus or node).

The latter point is an interesting discovery of our analysis, countering one’s intuition that two saddle modes may be easier to stabilize than a saddle and either an unstable focus or and unstable node. While an unstable focus and an unstable node have no principal stable direction–both the eigenvalues have positive real parts – they bring about rotation in the phase space. Operating in the antiresonance frequency bands allows for such rotation to explore the stable principal direction of the saddle mode, along which oscillations are tamed.

The insight gathered from the application of the theory of motion in a rapidly oscillating field has potential ramifications beyond stabilization of systems without any stable features. In fact, our analysis suggests that even a small, positive damping modulation could reverse the benefits of stiffness modulation. When the damping and stiffness modulations are out-of-phase, the effective damping of the oscillator decreases, potentially to negative values. This observation points to a previously overlooked issue in the application of Kapitza’s principles. Whether these principles are applied to reverse gravity in liquids^15^ or to stabilize non-Foster circuits^16^, the designer makes the assumption of energy conservation or, at most, of constant damping. We warn prudence against such an assumption, especially when the operating conditions of the system are uncertain, and there is limited knowledge about its full dynamics.

The experimental demonstration of the theoretically predicted antiresonant response is a key result of our work. Our experimental research also brings about: (i) a unique example of a mechanical oscillator that is designed to display both negative stiffness and negative damping, through snap-through and aeroelastic instabilities, (ii) the first mechanical example of a stable system switching between two unstable modes (a saddle and an unstable focus), and (iii) the use of fluid-structure interactions and fluid-induced instabilities to elicit unconventional behavior in mechanical systems^45,46^. Our mechanical oscillator has the potential of becoming a textbook example of dynamic stabilization, integrating different mechanical engineering disciplines within a broader dynamical systems perspective.

Our work is not free of limitations. From a theoretical point of view, our analysis is limited to linear stability. Developing a nonlinear model of the mechanical oscillator may shed further light on dynamic stabilization; for example, a nonlinear model may help predict RMS values outside of the antiresonance window. We also acknowledge that the application of the theory of motion in a rapidly evolving field is not adequate to fully capture the stability landscape of the modified Meissner’s oscillator, echoing recommendations by Berg and Wickramasinghe^23,25^ on the use of stability maps for the study of Hill’s equation. From an experimental perspective, the main limitation is that the air flow created by our fan is not spatially uniform, likely affecting the galloping response of the oscillator. Moving to a more uniform air flow could allow for a better measurement of the flow-induced negative damping parameter. One could also think of replacing the flow with a dry friction-induced follower force, also known to generate flutter-like instabilities^47^. Another alternative may entail the use of piezoelectric materials, with active shunting circuits^48^. Using switching resistors attaining negative resistances (for example, through negative impedance converters^49^), one may induce damping modulation in the absence of any incoming flow. The use of electric circuits can be further expanded, leading to a fully electrical realization of the proposed oscillator by including a switching resistor in the design by Alex-Amor et al.^16^.

Overall, our work opens the door to the stabilization of systems that do not display any stable feature, through open-loop modulation of physical properties in compact frequency bands. We foresee several application domains that could be transformed by this discovery, including robotics, structural engineering, metamaterials, and network theory. In robotics, stabilization of unstable systems via switching could be leveraged for open-loop controlled gait and locomotion–something that could allow to preserve on-board computational resources for other, more critical functions. This is particularly relevant for biped locomotion, which is often described through an inverted pendulum^50^. Whether it is running in a fluid or maneuvering onboard a ship or a train, our approach promises the stabilization of extreme gait patterns in dynamic environments through open-loop modulation of stiffness and damping. In structural engineering, the prospect of stabilizing a structure on the brink of collapse (with an unstable average) by switching between unstable configurations is enticing. An engineer could think of creating reticulated structures (trusses or frames) with switching structural elements, which can be turned on or off to connect/disconnect nodes of the structure. This modulation of stiffness and damping may be particularly helpful for the stabilization of bridges that experience negative damping due to pedestrians’ compound motion^20^ or wind loading^19^. With respect to metamaterials, there is a growing body of research pointing at the possibility of leveraging the time-modulation of physical parameters for the design of non-reciprocal media^51–54^, where the flow of energy differs along different directions. In this context, parameters are typically chosen to avoid instabilities; this design choice can be relaxed upon embracing the principles unveiled by our work, potentially unlocking the potential of non-reciprocal media. Last, our work could also help shed light on the origin of windows of opportunity in switching networks of chaotic dynamical systems^55,56^. It has been observed that switching networks of some chaotic systems exhibit synchronous dynamics in compact frequency bands, but the origins of this phenomenon have remained elusive. Our work offers a different lens through which one may explore the emergence of these windows, as an orchestrated, dynamic interplay between unstable stables of different type, saddles, unstable foci, and unstable nodes.

## Methods

### Kapitza’s pendulum

Linearizing in the vicinity of the vertical upward position, the dynamics of an inverted pendulum is described by the following equation^5^:

6 $$\ddot{x}(t)+\frac{g}{\ell }\left(-1-\frac{\ddot{y}(t)}{g}\right)x(t)=0.$$

In the standard form of the model, the vertical oscillation is assumed to be harmonic ($y(t)=Y\cos (\omega t)$, with *Y* being the amplitude and *ω* the radian frequency of the base excitation), so that the pendulum is described by Mathieu’s equation^7^.

Dynamic stabilization of Kapitza’s pendulum is typically studied through the theory of motion in a rapidly varying field^5^. Assuming $\omega \, \gg \, \sqrt{\frac{g}{\ell }}$ (the rate at which the pendulum would depart from its upward equilibrium without base excitation), this theory allows one to decompose the dynamics into a fast, small, and zero-mean oscillation, *ξ*(*t*), and a slow, smooth motion, *X*(*t*). Specifically, we replace *x*(*t*) = *ξ*(*t*) + *X*(*t*) into (6),

7 $$\ddot{X}(t)+\ddot{\xi }(t)+\frac{g}{\ell }\left(-1-\frac{\ddot{y}(t)}{g}\right)X(t)+\frac{g}{\ell }\left(-1-\frac{\ddot{y}(t)}{g}\right)\xi (t)=0.$$

This equation involves fast and slow terms, which shall be separated. For the fast term, we simply set

8 $$\ddot{\xi }(t)=\frac{X(t)}{\ell }\ddot{y}(t),$$

given that all the other terms are either slow (they have *X*(*t*)) or small (they have *ξ*(*t*)). Integrating (8) for a harmonic base excitation, we determine $\xi (t)=\frac{X(t)}{\ell }y(t)$. Next, we replace for *ξ*(*t*) in (7) and compute the time-average,

9 $$\ddot{X}(t)+\frac{g}{\ell }\left(-1+\frac{{Y}^{2}{\omega }^{2}}{2\ell g}\right)X(t)=0.$$

Here, we used the fact that the average of the product between the base acceleration and the base motion, $\overline{y(t)\ddot{y}(t)}$, is $\frac{{Y}^{2}{\omega }^{2}}{2}$. Thus, for a given base excitation *Y*, one can determine a sufficiently large value of the oscillation frequency *ω* such that $\frac{{Y}^{2}{\omega }^{2}}{2\ell g} > 1$ for the effective potential to be a quadratic well (that is, for the effective stiffness to be positive). Note that an equivalent line of arguments can be carried out for a different type of modulation, like a square wave, by only retaining the fundamental harmonic of the modulation in (9).

Until this point, this material is from the classical textbook of Landau and Lifshitz^5^. What is less studied, or at least not made obvious in textbooks, is the implication of dynamic stabilization on the time-varying coefficient of the governing equation^57^. We isolate the term in parentheses in (6), which corresponds to the non-dimensional, time-varying stiffness of the pendulum, $\kappa (t)=-1+\frac{{\omega }^{2}Y}{g}\cos (\omega t)$. Such a stiffness has a negative mean (−1) and oscillates between ${\kappa }_{\pm }=-1\pm \frac{{\omega }^{2}Y}{g}$. For dynamic stabilization to be successful, $\omega \, \gg \, \sqrt{\frac{g}{\ell }}$ and $\frac{{Y}^{2}{\omega }^{2}}{2\ell g} > 1$ so that *κ*_+_ ≫ 1 and *κ*_−_ ≪ − 1. Thus, the base excitation must create a restoring force over time for a substantial portion of the oscillation cycle. It is such a stabilizing effect that supports the mechanism of dynamic stabilization.

### Derivation of (3)

Here, we follow similar steps to those reported above to describe Kapitza’s pendulum through the theory of motion in a rapidly evolving field^5^. By replacing *x*(*t*) = *ξ*(*t*) + *X*(*t*) into (1), we obtain

10 $$\ddot{X}(t)+	 \ddot{\xi }(t)+\frac{{a}_{c}}{m}\left(1+\frac{{b}_{c}}{{a}_{c}}p(t)\right)\left(\dot{X}(t)+\dot{\xi}(t)\right) \\ +	 \frac{{a}_{k}}{m}\left(1+\frac{{b}_{k}}{{a}_{k}}p(t)\right)\left(X(t)+\xi (t)\right)=0,$$

where we approximate the square-wave modulation as a harmonic function $p(t)\simeq P\cos (\omega t+\phi )$, where *ϕ* is some phase angle – for example, $\phi=\frac{\pi }{2}$ for signal (2) with $\delta=\frac{1}{2}$. Isolating the fast terms, we obtain

11 $$\ddot{\xi }(t)+\left(\frac{{b}_{c}}{m}\dot{X}(t)+\frac{{b}_{k}}{m}X(t)\right)p(t)=0,$$

where we retained only the highest order derivative in time. We can solve for *ξ*(*t*),

12 $$\xi (t)=\left(\frac{{b}_{c}}{m}\dot{X}(t)+\frac{{b}_{k}}{m}X(t)\right)\frac{P}{{\omega }^{2}}\cos (\omega t+\phi ).$$

Next, we substitute (12) in (10) and take an average in time to obtain

13 $$\ddot{X}(t)+\frac{{a}_{c}}{m}\dot{X}(t)+\frac{{b}_{c}}{m}\overline{p(t)\dot{\xi}(t)}+\frac{{a}_{k}}{m}X(t)+\frac{{b}_{k}}{m}\overline{p(t)\xi (t)}=0.$$

By noting that $\overline{p(t)\dot{\xi}(t)}=0$ and that $\overline{p(t)\xi (t)}=\left(\frac{{b}_{c}}{m}\dot{X}(t)+\frac{{b}_{k}}{m}X(t)\right)\frac{{P}^{2}}{2{\omega }^{2}}$, we retrieve (3).

### Proof of conditions (4)

Proving the two conditions in (4) requires us to separately consider the two cases of *ρ*_1_ and *ρ*_2_ complex conjugate (case a) or both real (case b). For case a, ${\rho }_{1}={\rho }_{2}^{\star }$ (a superimposed star indicates complex conjugation), so that $\det \Phi (T)=| {\rho }_{1}{| }^{2}$ and $\Phi (T)=2{{{\rm{Re}}}}\left({\rho }_{1}\right)$, where “${{{\rm{Re}}}}$” is the real part. In this case, $\det \Phi (T) < 1$ if and only if the eigenvalues are in the unit circle and $| {{{\rm{tr}}}}\Phi (T)| < 1+\det \Phi (T)$ by construction. For case b, we shall map the unit square in the *ρ*_1_ − *ρ*_2_ plane into a stability domain in the ${{{\rm{tr}}}}\Phi (T)-\det \Phi (T)$ plane. The boundaries of the transformed square are obtained by setting one of the eigenvalues, say the first one, to  ± 1, which implies ${\rho }_{2}=\pm \det \Phi (T)$ and $\pm 1+{\rho }_{2}={{{\rm{tr}}}}\Phi (T)$, that is, ${{{\rm{tr}}}}\Phi (T)=\pm \left(1+\det \Phi (T)\right)$. The other boundary of the domain is $\det \Phi (T)=1$, corresponding to *ρ*_1_ = *ρ*_2_ = ± 1.

### Stability analysis for two saddles

We focus on condition (4b) for (5b), as condition (4a) is automatically satisfied by (5a) assuming *a*_*c*_ > 0. In the case of the saddles, both Δ_1_ and Δ_2_ are positive. Let us start with the right-hand-side of the inequality: at *T* = 0, it is equal to 2, its slope is $-\frac{{a}_{c}}{m}$, and its curvature is $\frac{{a}_{c}^{2}}{{m}^{2}}$; as *T* increases, the function exponentially decays to one at the (negative) rate of $-\frac{{a}_{c}}{m}$. The function on the left-hand-side of the inequality has the same value and slope at *T* = 0 as the term on the right-hand-side. The curvature is, instead, given by

14 $$\frac{{a}_{c}^{2}}{{m}^{2}}-\frac{2 a_k}{m},$$

which is larger than $\frac{{a}_{c}^{2}}{{m}^{2}}$ since both modes are saddles. Likewise, as *T* increases, the function exponentially grows to infinity at the (positive) rate of $\frac{1}{2}\left[(1-\delta )\left(\sqrt{{\Delta }_{1}}-\frac{c_1}{m}\right)+\delta \left(\sqrt{{\Delta }_{2}}-\frac{c_2}{m}\right)\right]$. Thus, while the two functions start at the same value and with the same slope, the initial upward curvature of the left-hand-side is strong enough to ensure their separation as *T* increases, hence instability.

### Experimental setup

The beam is a 4 cm  × 12 cm rectangle created by laser cutting a 1.3 mm-thick PETG sheet (McMaster-Carr 9513K92). The tip mass is 3D-printed with Ultimaker PLA at 50% infill with a gyroid pattern. A 10 mm deep cut is printed on one semi-circular face of the tip mass; we then insert and glue the beam into this cutout. On the opposite semi-circular face, a 10 mm-diameter, 2 mm-deep cylinder cutout is made to facilitate the positioning of the 10 mm-diameter neodymium permanent magnets, which are then glued to the tip mass. We use two magnets in series to provide a stronger interaction with the magnetic coil. The combined mass of the cylinder and the two magnets is *m* = 49.00 g, which is much larger than the beam mass (6.56 g). The cantilever beam is clamped with two L-shaped brackets, as shown in Supplementary Fig. 2a, so that its effective length is 10 cm. The beam is clamped to a moving micrometer-precision stage and fixed to a 1 inch framing rail, which is attached to a breadboard plate to create a compact, movable setup that can be transferred from the optical table (for vibration measurement) to a UTS (for stiffness measurement). We then attach the magnetic coil (APW FC-6489) to a bracket, which is also fixed to the same aluminum extrusion and adjusted such that the permanent magnets are 10.0 mm away from the face of the magnetic coil. The coil is aligned to the center of the permanent magnet using a custom-made positioning device for planar movement, shown in Supplementary Fig. 2b. To create the air flow necessary to trigger galloping instabilities, a 12 V, 120 mm-diameter fan (Mechatronics MS1238E12B-FHR-2EM) is placed 39.5 mm above the rectangular face of the tip mass, using a vertical positioning guide. The fan is also aligned using a ruler-compass custom positioning device, shown in Supplementary Fig. 2c, d. The fan is positioned at  − 45^o^ from the direction aligned with the tip beam at rest, and at 45.7 mm from the center of the vertical positioning guide. Measurements are carried out with room relative humidity recorded at approximately 30%. The velocity of the tip mass is recorded using a laser doppler vibrometer (Polytec VibroFlex Connect VFX-F-110 Front-End and Neo VFX-I-110 Sensor Head). A retro-reflective tape strip is placed onto the tip mass to improve the signal quality. The signal from the vibrometer is read by an oscilloscope. All post-processing is performed in MATLAB.

### Switching circuit and synchronization

A schematic of our switching circuit is provided in Supplementary Fig. 3. To support the shutter mechanism, we create a custom support made of laser-cut acrylic and framing rails. The shutter is a flat disk in which two opposite quarters are removed. As it rotates, the shutter periodically blocks the wind produced by the fan. Its diameter is 360 mm at the widest point and its thickness is a 1.5 mm. The shutter is powered by a Dynamixel MX430-T150-BB servomotor attached to a gear system with a 1:2 ratio. The Dynamixel motor is controlled by an Arduino Uno R3 with a Dynamixel Shield attached on top, with 12 V being supplied to the Shield. The fan is powered by a power supply; we determine 5.3 V to be the optimal voltage to be given to the fan (which can take 12 V maximum), and this results in a wind field varying from 1.1 to 1.7 m/s where the tip mass is located (measured with a manual anemometer). To control the magnetic coil, the Arduino supplies a signal to a Sunfounder 4-Channel Relay board, causing it to periodically open and close its circuit. A wire is connected to one of the relays which, when closed, supplies 24 V to the coil. To synchronize the shutter and the magnetic coil so that the shutter covers the beam when the coil is on and vice versa, we need to determine the periods and timing parameters that keep the shutter and magnet in sync with each other. The system is controlled by the central Arduino Uno R3 which requires an RPM value to run the motor, and a time quantity (half of the switching period) to wait between switching the relay on and off. To determine the right RPM for each period, we tape an opaque material (painter’s tape) along the circumference of the shutter and we point the laser vibrometer at the shutter, as shown in Supplementary Fig. 4a. A voltage probe is also attached to the two wires leading to the electromagnet, as shown in Supplementary Fig. 4b. During system operation, as the laser hits the opaque material, the oscilloscope reads a flat line from the vibrometer measurement; conversely, when the laser detects no shutter, the signal becomes noisy. This is shown by the yellow signal in Supplementary Fig. 4c. Similarly, as the relay is opened and closed, the voltage drop measured across the magnet goes from 0 to some nominal voltage, seen in blue in Supplementary Fig. 4c. For a range of switching periods from 159 to 268 ms, at intervals of 8–12 ms ( ≈ 10 ms), we identify specific periods and RPM values such that the shutter and coil are synchronized, as shown in Supplementary Fig. 4c.

### Negative damping estimation

The response of the beam to the air flow shows upward trends, but also features fluctuations that make the application of the logarithmic decrement procedure challenging. To overcome this issue, we apply a kernel-based smoothing algorithm to the data. The weights of a kernel are evaluated as ${w}_{i}^ \ast=\frac{1}{| q-i|+1},{w}_{i}=\frac{{w}_{i}^*}{\sum {w}_{i}^*}$, where *i* = 1,  … *n* represents an index of the kernel, *n* is the size of the kernel and *q* is the central index. After a trial-and-error process, we determine that a kernel size of *n* = 9 and three iterations of smoothing are most effective. Regions of interest are selected as those where the instantaneous slopes of the data points are at least 10% of the maximum observed slope. We then apply a linear *polyfit* to the original data points within this region, and we take the slope of the trend line to calculate the damping ratio within that region, using the formula: $\xi=\,{{{\rm{slope}}}}/\sqrt{4{\pi }^{2}+{{{{\rm{slope}}}}}^{2}}$.

### Force measurement

The experimental setup for stiffness testing is shown in Figs. 3b and S5a. We perform experiments in displacement-control with a UTS (Instron 5982 with a 100 N load cell, operated at a rate of 1 mm/s). The detail in Supplementary Fig. 5b shows a close-up of the custom gripper used to subject the beam to a tip displacement without over-constraining it. The gripper features a U-shaped bracket with two holes, onto which we slide a metallic pin. The metallic pin also slides through the hole located at the center of the tip mass, and is kept in place using small cutouts of a silicone rubber tube. Turning the magnetic coil on and increasing the supplied voltage allow the system to evolve from a monostable to a bistable energy landscape, causing the stiffness around the rest position to change continuously from positive to negative, as shown in Supplementary Fig. 5c.

## Supplementary information

Supplementary Information

Description of Additional Supplementary Files

Supplementary Video 1

Supplementary Video 2

Supplementary Video 3

Supplementary Video 4

Supplementary Video 5

Supplementary Video 6

Supplementary Video 7

Supplementary Video 8

Supplementary Video 9

Supplementary Video 10

Supplementary Video 11

Supplementary Video 12

Supplementary Video 13

Transparent Peer Review file

## Source data

Source Data

## Data Availability

All experimental data generated in this study is provided with this article as a Source Data folder. Source data are provided with this paper.

## References

[1] Turner, A. J. The time museum. vol. i: Time measuring instruments. part 3: Water-clocks, sand-glasses, fire-clocks. *The Time Museum. Vol. I: Time measuring instruments. Part 3: Water-clocks* (The Time Museum, 1984).

[2] Rosheim, M. *Leonardo’s Lost Robots* (Springer, 2006).

[3] Lewis, F. Introduction to modern control theory. *Appl. Optimal Control Estimation* 3–24 (1992).

[4] Kapitza, P. L. A pendulum with oscillating suspension. *Uspekhi Fizicheskikh Nauk* **44**, 7–20 (1951).

[5] Landau, L. D. & Lifshitz, E. M.*Course of Theoretical Physics* (Elsevier, 2013).

[6] Meissner, E. Ueber schüttelerscheinungen in systemen mit periodisch veränderlicher elastizität. *Schweizerische Bauzeitung* **72**, 95–98 (1918).

[7] Mathieu, É Mémoire sur le mouvement vibratoire d’une membrane de forme elliptique. *J. de Mathé. Pures Appl.* **13**, 137–203 (1868).

[8] Magnus, W. & Winkler, S.*Hill’s Equation* (Courier Corporation, 2004).

[9] Smith, H., Blackburn, J. & Grnbech-Jensen, N. Stability and hopf bifurcations in an inverted pendulum. *Am. J. Phys.* **60**, 903–908 (1992).

[10] Kovacic, I., Rand, R. & Mohamed Sah, S. Mathieu’s equation and its generalizations: overview of stability charts and their features. *Appl. Mech. Rev.* **70**, 020802 (2018).

[11] Sharma, A. & Sinha, S. On instability pockets and influence of damping in parametrically excited systems. *J. Vibration Acoust.* **140**, 051001 (2018).

[12] Levi, M. Geometrical aspects of rapid vibrations and rotations. *Philos. Trans. R. Soc. A* **377**, 20190014 (2019).

[13] Paul, W. Electromagnetic traps for charged and neutral particles. *Rev. Modern Phys.* **62**, 531 (1990).

[14] Taha, H. E., Kiani, M., Hedrick, T. L. & Greeter, J. S. Vibrational control: a hidden stabilization mechanism in insect flight. *Sci. Robot.* **5**, eabb1502 (2020). 32999048 10.1126/scirobotics.abb1502

[15] Apffel, B., Novkoski, F., Eddi, A. & Fort, E. Floating under a levitating liquid. *Nature* **585**, 48–52 (2020). 32879504 10.1038/s41586-020-2643-8

[16] Alex-Amor, A., Ptitcyn, G. & Engheta, N. Kapitza-inspired stabilization of non-Foster circuits via time modulations. *Phys. Rev. Appl.* **24**, 024022 (2025).

[17] Wang, Z. & Safavi-Naeini, A. H. Quantum control and noise protection of a Floquet 0-*π* qubit. *Phys. Rev. A* **109**, 042607 (2024).

[18] Thompson, D.*Railway Noise and Vibration: Mechanisms, Modelling and Means of Control* (Elsevier, 2008).

[19] Beards, C.*Structural Vibration: Analysis and Damping* (Elsevier, 1996).

[20] Belykh, I. et al. Emergence of the london millennium bridge instability without synchronisation. *Nat. Commun.* **12**, 7223 (2021). 34893627 10.1038/s41467-021-27568-y PMC8664840

[21] Rugh, W. J. *Linear System Theory* (Prentice-Hall, Inc., 1996).

[22] Carlos, F. & Joaquin, C. On periodic differential equations with dissipation. *Electron. J. Qual. Theory Differ. Equ.* **2018**, 1–17 (2018).

[23] Wickramasinghe, I. & Berg, J. M. Vibrational control of Mathieu’s equation. In *2013 IEEE/ASME International Conference on Advanced Intelligent Mechatronics*, 686–691 (IEEE, 2013).

[24] Arnol’d, V. I. Remarks on the perturbation theory for problems of mathieu type. *Russian Math. Surveys* **38**, 215 (1983).

[25] Berg, J. M. & Wickramasinghe, I. M. Vibrational control without averaging. *Automatica* **58**, 72–81 (2015).

[26] DeCarlo, R. A., Branicky, M. S., Pettersson, S. & Lennartson, B. Perspectives and results on the stability and stabilizability of hybrid systems. *Proc. IEEE* **88**, 1069–1082 (2000).

[27] Sirohi, J. & Mahadik, R. Harvesting wind energy using a galloping piezoelectric beam. *J. Vibration Acoust.* **134**, 011009 (2012).

[28] Barrero-Gil, A., Alonso, G. & Sanz-Andres, A. Energy harvesting from transverse galloping. *J. Sound Vibration* **329**, 2873–2883 (2010).

[29] Abdelkefi, A., Hajj, M. & Nayfeh, A. Piezoelectric energy harvesting from transverse galloping of bluff bodies. *Smart Mater. Struct.* **22**, 015014 (2012).

[30] Cottone, F., Vocca, H. & Gammaitoni, L. Nonlinear energy harvesting. *Phys. Rev. Lett.* **102**, 080601 (2009). 19257728 10.1103/PhysRevLett.102.080601

[31] Daqaq, M. F., Masana, R., Erturk, A. & Dane Quinn, D. On the role of nonlinearities in vibratory energy harvesting: a critical review and discussion. *Appl. Mech. Rev.* **66**, 040801 (2014).

[32] Churchill, C. B., Shahan, D. W., Smith, S. P., Keefe, A. C. & McKnight, G. P. Dynamically variable negative stiffness structures. *Sci. Adv.* **2**, e1500778 (2016). 26989771 10.1126/sciadv.1500778 PMC4788489

[33] Sames, C. et al. Antiresonance phase shift in strongly coupled cavity qed. *Phys. Rev. Lett.* **112**, 043601 (2014). 24580448 10.1103/PhysRevLett.112.043601

[34] Yang, F. et al. Mechanically modulated sideband and squeezing effects of membrane resonators. *Phys. Rev. Lett.* **127**, 184301 (2021). 34767395 10.1103/PhysRevLett.127.184301

[35] Fokoua, E. N., Mousavi, S. A., Jasion, G. T., Richardson, D. J. & Poletti, F. Loss in hollow-core optical fibers: mechanisms, scaling rules, and limits. *Adv. Opt. Photonics* **15**, 1–85 (2023).

[36] Lei, P., Xu, M., Bai, Y., Chen, Z. & Xie, X. Anti-resonant acoustic waveguides enabled tailorable brillouin scattering on chip. *Nat. Commun.* **15**, 3877 (2024). 38719846 10.1038/s41467-024-48123-5 PMC11078926

[37] Ormondroyd, J. & Den Hartog, J. The theory of the dynamic vibration absorber. *J. Fluids Eng.* **49**, 021007 (1928).

[38] Tondl, A. To the problem of self-excited vibration suppression. *Eng. Mech.* **15**, 297–307 (2008).

[39] Dohnal, F. General parametric stiffness excitation–anti-resonance frequency and symmetry. *Acta Mech.* **196**, 15–31 (2008).

[40] Karev, A. & Hagedorn, P. Global stability effects of parametric excitation. *J Sound Vibration* **448**, 34–52 (2019).

[41] Mailybaev, A. A. & Seyranian, A. P. Stabilization of statically unstable systems by parametric excitation. *J Sound Vibration* **323**, 1016–1031 (2009).

[42] Kassakian, J. G., Schlecht, M.F. & Verghese, G.C. *Principles of Power Electronics* (Addison-Wesley, 1991).

[43] Belykh, I., Di Bernardo, M., Kurths, J. & Porfiri, M. Evolving dynamical networks. *Phys. D: Nonlinear Phenom.* **267**, 1–6 (2014).

[44] Porfiri, M., Jeter, R. & Belykh, I. Antiresonance in switched systems with only unstable modes. *Phys. Rev. Res.* **3**, L022001 (2021).

[45] Casadei, F. & Bertoldi, K. Harnessing fluid-structure interactions to design self-regulating acoustic metamaterials. *J. Appl. Phys.* **115**, 034907 (2014).

[46] Bigoni, D. et al. Flutter instability in solids and structures, with a view on biomechanics and metamaterials. *Proc. R. Soc. A* **479**, 20230523 (2023).

[47] Bigoni, D. & Noselli, G. Experimental evidence of flutter and divergence instabilities induced by dry friction. *J. Mech. Phys. Solids* **59**, 2208–2226 (2011).

[48] Hagood, N. W. & Von Flotow, A. Damping of structural vibrations with piezoelectric materials and passive electrical networks. *J. Sound Vibration* **146**, 243–268 (1991).

[49] Chen, Y., Rangarajan, G. & Ding, M. General stability analysis of synchronized dynamics in coupled systems. *Phys. Rev. E* **67**, 026209 (2003). 10.1103/PhysRevE.67.026209 12636778

[50] Hemami, H. & Golliday Jr, C. The inverted pendulum and biped stability. *Mathe. Biosci.* **34**, 95–110 (1977).

[51] Swinteck, N. et al. Bulk elastic waves with unidirectional backscattering-immune topological states in a time-dependent superlattice. *J. Appl. Phys.* **118**, 063103 (2015).

[52] Wang, Y. et al. Observation of nonreciprocal wave propagation in a dynamic phononic lattice. *Phys. Rev. Lett.* **121**, 194301 (2018). 30468594 10.1103/PhysRevLett.121.194301

[53] Trainiti, G. et al. Time-periodic stiffness modulation in elastic metamaterials for selective wave filtering: theory and experiment. *Phys. Rev. Lett.* **122**, 124301 (2019). 30978089 10.1103/PhysRevLett.122.124301

[54] Attarzadeh, M., Callanan, J. & Nouh, M. Experimental observation of nonreciprocal waves in a resonant metamaterial beam. *Phys. Rev. Appl.* **13**, 021001 (2020).

[55] Jeter, R. & Belykh, I. Synchronization in on-off stochastic networks: windows of opportunity. *IEEE Trans Circ. Syst. I: Regular Papers* **62**, 1260–1269 (2015).

[56] Golovneva, O., Jeter, R., Belykh, I. & Porfiri, M. Windows of opportunity for synchronization in stochastically coupled maps. *Phys D: Nonlinear Phenom* **340**, 1–13 (2017).

[57] Grandi, A. A. *New physical insights on time periodic systems: from extreme parametric resonance to synchronized dynamic stabilization*. Ph.D. thesis, Sorbonne Université (2022).

