# Supplementary material for: Dynamic stabilization of a mechanical oscillator in the absence of any stable feature
Source: Nat Commun. 2026 Mar 10;17:6024. doi: 10.1038/s41467-026-70493-1 (PMC13346612; doi:10.1038/s41467-026-70493-1)
Supplement: Supplementary file 1 — Supplementary Information [file 41467_2026_70493_MOESM1_ESM.pdf]

Supplementary Information: "Dynamic stabilization of a mechanical oscillator in the absence of any stable feature"

David Xiedeng,[1] Paolo Celli,[1,*] and Maurizio Porfiri[2,3,4,5,†]

[1]*Department of Civil Engineering, Stony Brook University, Stony Brook, NY 11794, USA*
[2]*Center for Urban Science and Progress, Tandon School of Engineering, New York University, Brooklyn, NY 11201, USA*
[3]*Department of Biomedical Engineering, Tandon School of Engineering, New York University, Brooklyn, NY 11201, USA*
[4]*Department of Civil, Urban and Environmental Engineering, Tandon School of Engineering, New York University, Brooklyn, NY 11201, USA*
[5]*Department of Mechanical and Aerospace Engineering, Tandon School of Engineering, New York University, Brooklyn, NY 11201, USA*

(Dated: March 8, 2026)

[*] paolo.celli@stonybrook.edu
[†] mporfiri@nyu.edu

SUPPLEMENTARY DISCUSSION

Supplementary Note 1. Numerical validation of the theory of motion in a rapidly oscillating field 3

Supplementary Note 2. Descriptions of supplementary videos 12

Supplementary Note 3. Complete switching experiments data and additional results 13

SUPPLEMENTARY FIGURES

1 **Validation of numerical predictions from the theory of motion in a rapidly evolving field**. (a–e) Comparison between predictions from the theory of Landau and Lifshitz (blue, equations (3) and (12)) and the exact solution of the modified Meissner's model (black, equations (1) and (2))). In all cases, we set $\delta = \frac{1}{2}$, $x(0) = 1$ (arbitrary units), $\dot{x}(0) = 0$, $m = 10\,\text{kg}$, $a_k = -0.1\,\text{N}\,\text{m}^{-1}$, $a_c = 0.7\,\text{N}\,\text{s}\,\text{m}^{-1}$, and $T = 0.95\,\text{s}$ (corresponding to $\omega = \frac{60}{\tau_0}$, with $\tau_0 = 10\,\text{s}$). (a) Accurate prediction of stable response in the absence of any stable feature ($b_k = 100\,\text{N}\,\text{m}^{-1}$ and $b_c = -1\,\text{N}\,\text{s}\,\text{m}^{-1}$). Theoretical predictions remain accurate upon reducing the stiffness modulation (b, $b_k = 5\,\text{N}\,\text{m}^{-1}$ and $b_c = -1\,\text{N}\,\text{s}\,\text{m}^{-1}$) or increasing the damping modulation (c, $b_k = 100\,\text{N}\,\text{m}^{-1}$ and $b_c = -2\,\text{N}\,\text{s}\,\text{m}^{-1}$). Theoretical predictions lose accuracy upon excessively increasing the damping (d, $b_k = 100\,\text{N}\,\text{m}^{-1}$ and $b_c = -5\,\text{N}\,\text{s}\,\text{m}^{-1}$) or stiffness (e, $b_k = 155\,\text{N}\,\text{m}^{-1}$ and $b_c = -1\,\text{N}\,\text{s}\,\text{m}^{-1}$) modulation. (f) Illustration of how the parameters chosen in the simulations in (a–e) fall within the stability map of our system (red: unstable; light blue: stable). Solid and empty markers represent cases where the exact solution and the theory of Landau and Lifshitz agree or disagree, respectively; dashed lines have the same meaning as in Fig. 1d of the main text. 4

2 **Details of the experimental setup**. (a) Wide view of the experimental setup, with labels indicating its various parts. (b) Detail of the magnetic coil positioning system. (c,d) Details of the fan positioning system. 5

3 **Schematic of our switching circuit**. The circuit is described in the Methods. 6

4 **Synchronization of the switching circuit**. (a) Laser vibrometer measurement on the shutter, needed to determine the correct RPM to obtain specific switching periods. (b) Measurement of the voltage across the coil. (c) Synchronization between the shutter (yellow signal) and the coil (blue signal). 7

5 **Experimental stiffness characterization**. (a) Experimental setup, where the midpoint of the tip mass is moved along its lateral DOF by a UTS system, via a custom gripper shown in (b). The gripper is designed not to constrain the rotation of the tip mass. (c) Complete data from the stiffness experiments, including the open circuit response and the response at coil voltages from 4 V to 24 V, showing how the system's stiffness can be tuned from positive to negative values. 8

6 **Complete data from the damping characterization experiments**. All subfigures feature a time trace, where peaks are highlighted by circular markers, and a logarithmic decrement plot, where the natural logarithm of the peak amplitude is plot against the peak number. (a–c) Free response experiments to measure the positive damping of the system. (d–f) Aerodynamic loading experiments to measure the negative damping of the system. 9

7 **System's dynamic response as 24 V are provided to the coil, with the air flow blocked by the shutter**. The time history of the lateral velocity is accompanied by photos of the beam deformation. 10

8 **Additional results on switching dynamics**. Experimental time-histories, experimental RMS plots, and illustrations of the transcendental equation in condition (4b) that defines the emergence of antiresonance for the switched system (gray: $|\,\text{tr}\Phi(T)\,|$ and black $1+\det\Phi(T)$), for the following switching scenarios: (a,b) $k_1 = -23.68$ N/m, $\xi_1 = 0.0031$, $k_2 = 10.74$ N/m, $\xi_2 = -0.0024$; (c,d) $k_1 = -23.68$ N/m, $\xi_1 = \xi_2 = 0.0031$, $k_2 = 10.74$ N/m; and (e,f) $k_1 = k_2 = 10.74$ N/m, $\xi_1 = 0.0031$, $\xi_2 = -0.0024$. 11

Supplementary Note 1. NUMERICAL VALIDATION OF THE THEORY OF MOTION IN A RAPIDLY OSCILLATING FIELD

Here, we examine the accuracy of the theory of motion in a rapidly evolving field in approximating the dynamics of the modified Meissner's model. Specifically, we compare predictions from the theory of motion in a rapidly evolving field in equations (3) and (12) against the exact solution of the modified Meissner's model in equations (1) and (2) (Supplementary Figure 1). Throughout the simulations, we set $\delta = \frac{1}{2}$ and choose initial conditions as $x(0) = 1$ (arbitrary units) and $\dot{x}(0) = 0$. With respect to the solution from the theory of motion in a rapidly evolving field, we consider both the fast and slow dynamics, thereby plotting $X(t) + \xi(t)$, where $X(t)$ is the solution of equation (3) and $\xi(t)$ is given by equation (12), with $\phi = \frac{\pi}{2}$ and $P = \frac{4}{\pi}$. Initial conditions for equation (3) are $X(0) = 1$ and $\dot{X}(0) = \frac{Pb_k}{m\omega - Pb_c}$ to match the initial conditions of the modified Meissner's model in equations (1) and (2).

We confirm that the theory of motion in a rapidly varying field is successful in predicting stabilization of the modified Meissner's model in the absence of any stable feature (Supplementary Figure 1a). In agreement with our expectations from the stability bounds in Fig. 1d, it is also accurate in predicting the loss of stability due to weaker stiffness modulation (Supplementary Figure 1b) and the resilience of the phenomenon against increases in the damping modulation (Supplementary Figure 1c). In agreement with our expectations from Fig. 1d and from the literature [1, 2], the theory loses accuracy if the stiffness (Supplementary Figure 1d) or damping modulation (Supplementary Figure 1e) become too strong. Specifically, when the stiffness modulation increases above a threshold value, the system becomes unstable even though theory suggests a stable response – an equivalent pitfall to those seen by Berg and Wickramasinghe [1, 2]. On the contrary, when the damping modulation increases above a threshold value, the theory erroneously suggests a loss of stability. An illustration of how the parameters chosen for the simulations fall within the stability map of our system is provided in Supplementary Figure 1f.

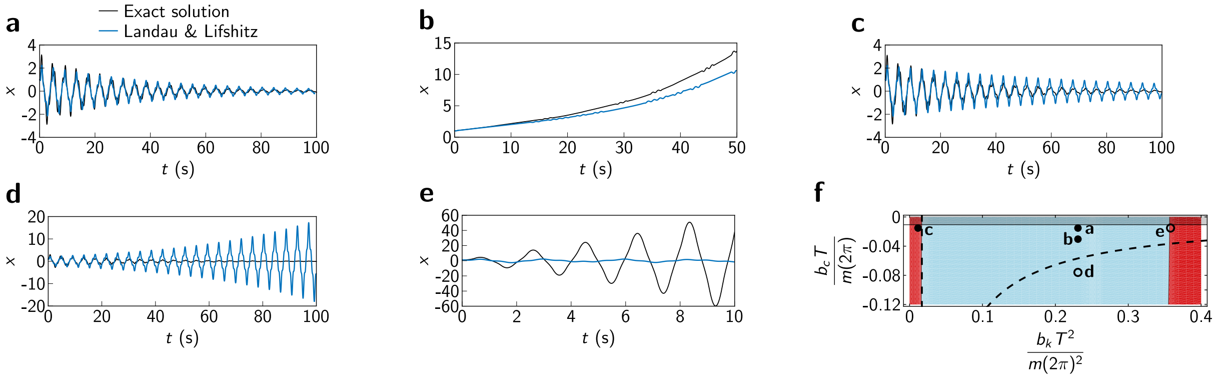

Supplementary Figure 1. **Validation of numerical predictions from the theory of motion in a rapidly evolving field**. (a–e) Comparison between predictions from the theory of Landau and Lifshitz (blue, equations (3) and (12)) and the exact solution of the modified Meissner's model (black, equations (1) and (2))). In all cases, we set $\delta = \frac{1}{2}$, $x(0) = 1$ (arbitrary units), $\dot{x}(0) = 0$, $m = 10\,\mathrm{kg}$, $a_k = -0.1\,\mathrm{N\,m^{-1}}$, $a_c = 0.7\,\mathrm{N\,s\,m^{-1}}$, and $T = 0.95\,\mathrm{s}$ (corresponding to $\omega = \frac{60}{\tau_0}$, with $\tau_0 = 10\,\mathrm{s}$). (a) Accurate prediction of stable response in the absence of any stable feature ($b_k = 100\,\mathrm{N\,m^{-1}}$ and $b_c = -1\,\mathrm{N\,s\,m^{-1}}$). Theoretical predictions remain accurate upon reducing the stiffness modulation (b, $b_k = 5\,\mathrm{N\,m^{-1}}$ and $b_c = -1\,\mathrm{N\,s\,m^{-1}}$) or increasing the damping modulation (c, $b_k = 100\,\mathrm{N\,m^{-1}}$ and $b_c = -2\,\mathrm{N\,s\,m^{-1}}$). Theoretical predictions lose accuracy upon excessively increasing the damping (d, $b_k = 100\,\mathrm{N\,m^{-1}}$ and $b_c = -5\,\mathrm{N\,s\,m^{-1}}$) or stiffness (e, $b_k = 155\,\mathrm{N\,m^{-1}}$ and $b_c = -1\,\mathrm{N\,s\,m^{-1}}$) modulation. (f) Illustration of how the parameters chosen in the simulations in (a–e) fall within the stability map of our system (red: unstable; light blue: stable). Solid and empty markers represent cases where the exact solution and the theory of Landau and Lifshitz agree or disagree, respectively; dashed lines have the same meaning as in Fig. 1d of the main text.

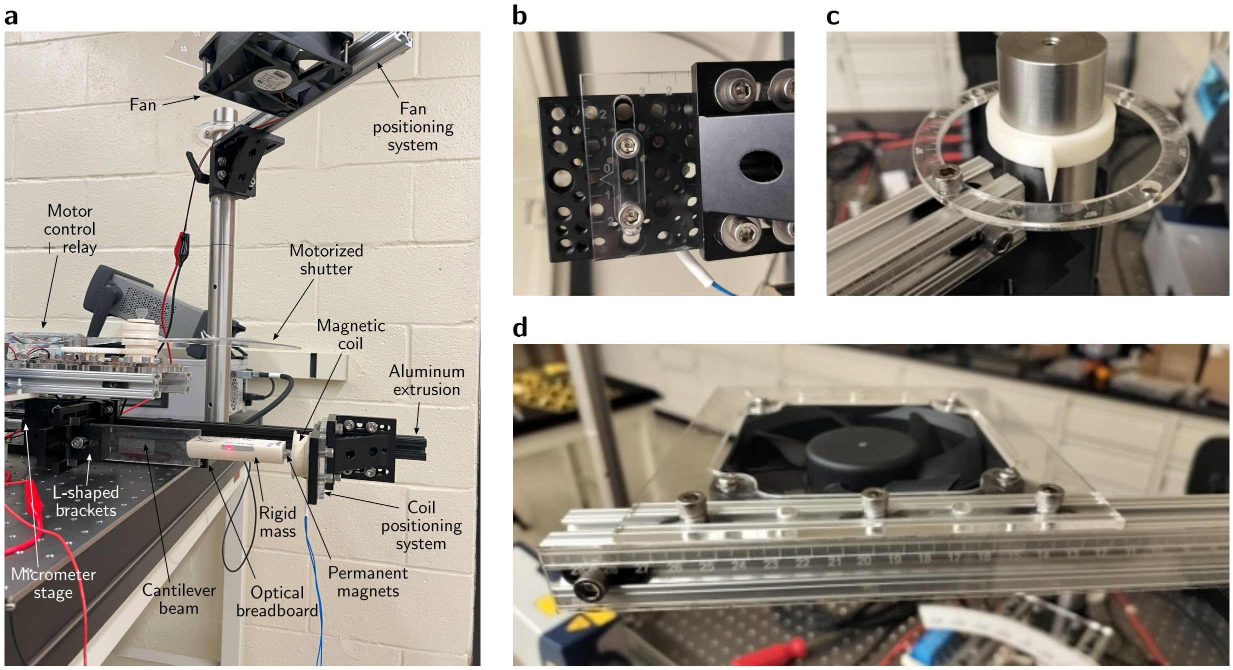

Supplementary Figure 2. **Details of the experimental setup**. (a) Wide view of the experimental setup, with labels indicating its various parts. (b) Detail of the magnetic coil positioning system. (c,d) Details of the fan positioning system.

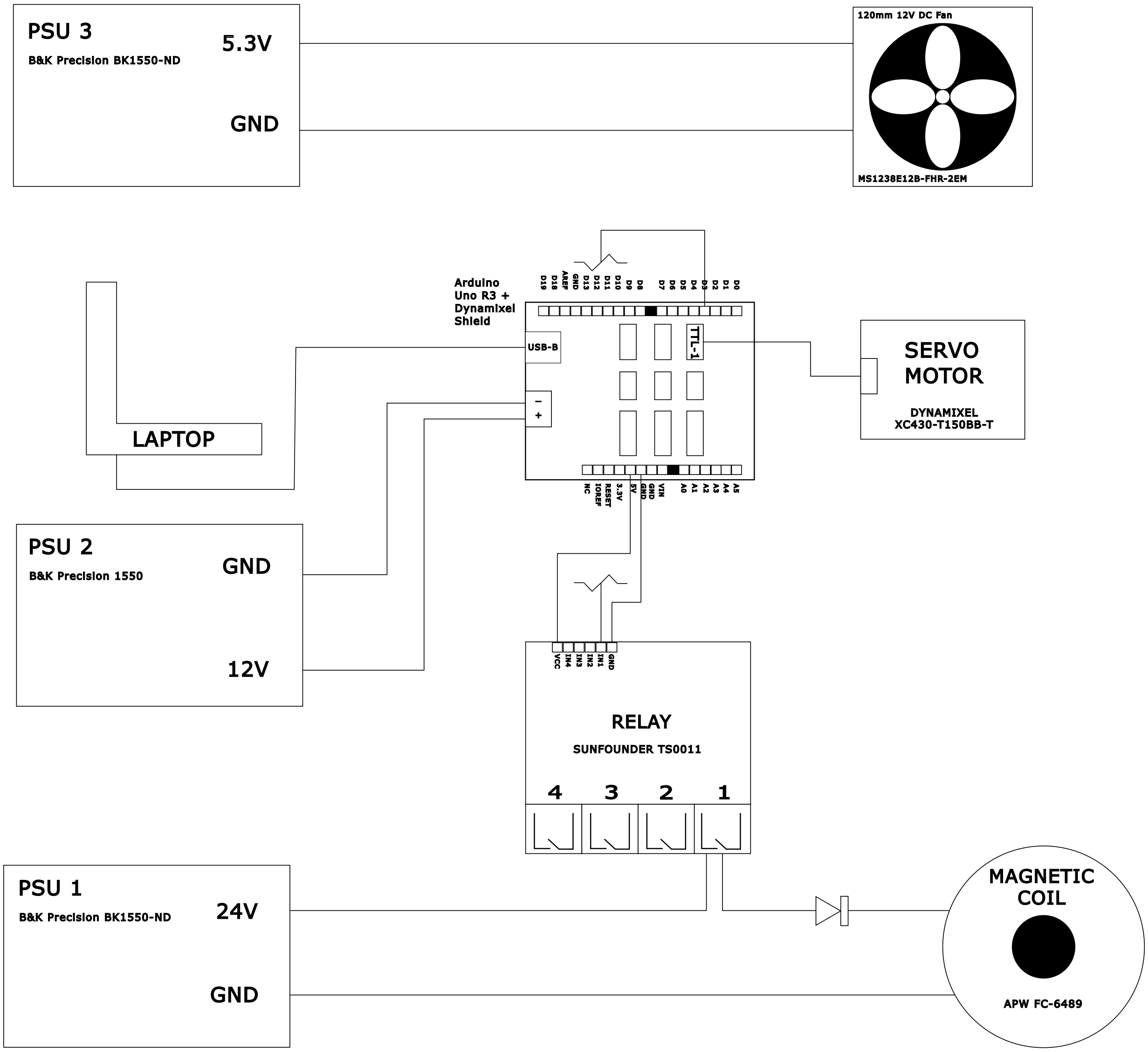

Supplementary Figure 3. **Schematic of our switching circuit**. The circuit is described in the Methods.

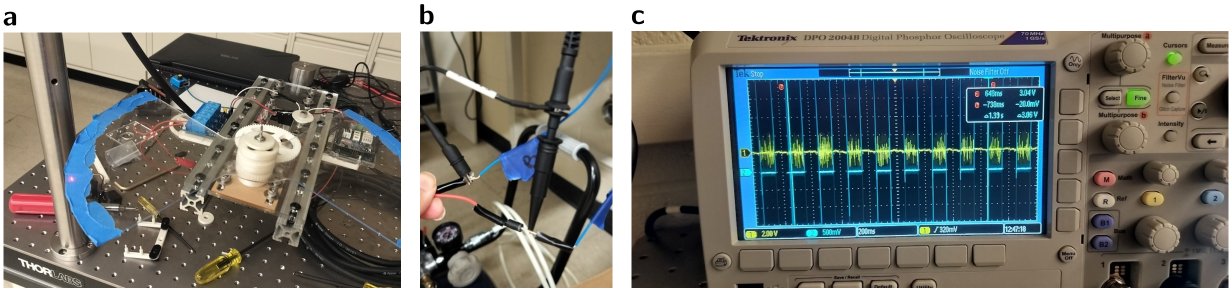

Supplementary Figure 4. **Synchronization of the switching circuit**. (a) Laser vibrometer measurement on the shutter, needed to determine the correct RPM to obtain specific switching periods. (b) Measurement of the voltage across the coil. (c) Synchronization between the shutter (yellow signal) and the coil (blue signal).

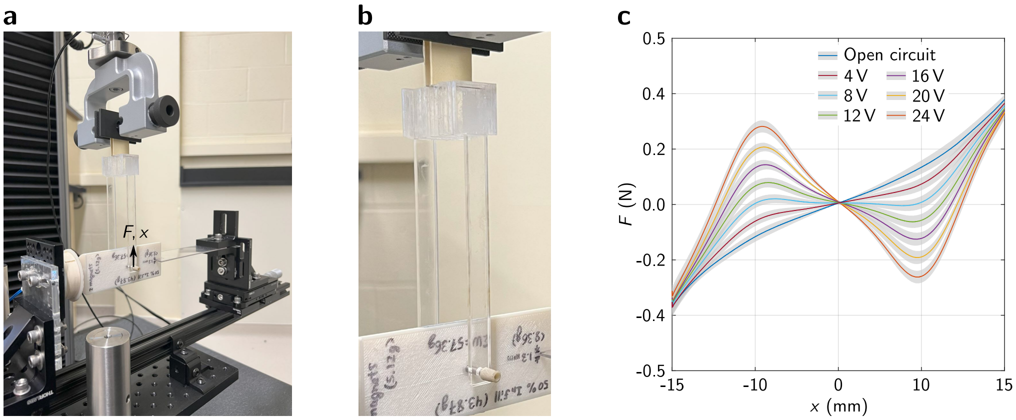

Supplementary Figure 5. **Experimental stiffness characterization**. (a) Experimental setup, where the midpoint of the tip mass is moved along its lateral DOF by a UTS system, via a custom gripper shown in (b). The gripper is designed not to constrain the rotation of the tip mass. (c) Complete data from the stiffness experiments, including the open circuit response and the response at coil voltages from 4 V to 24 V, showing how the system's stiffness can be tuned from positive to negative values.

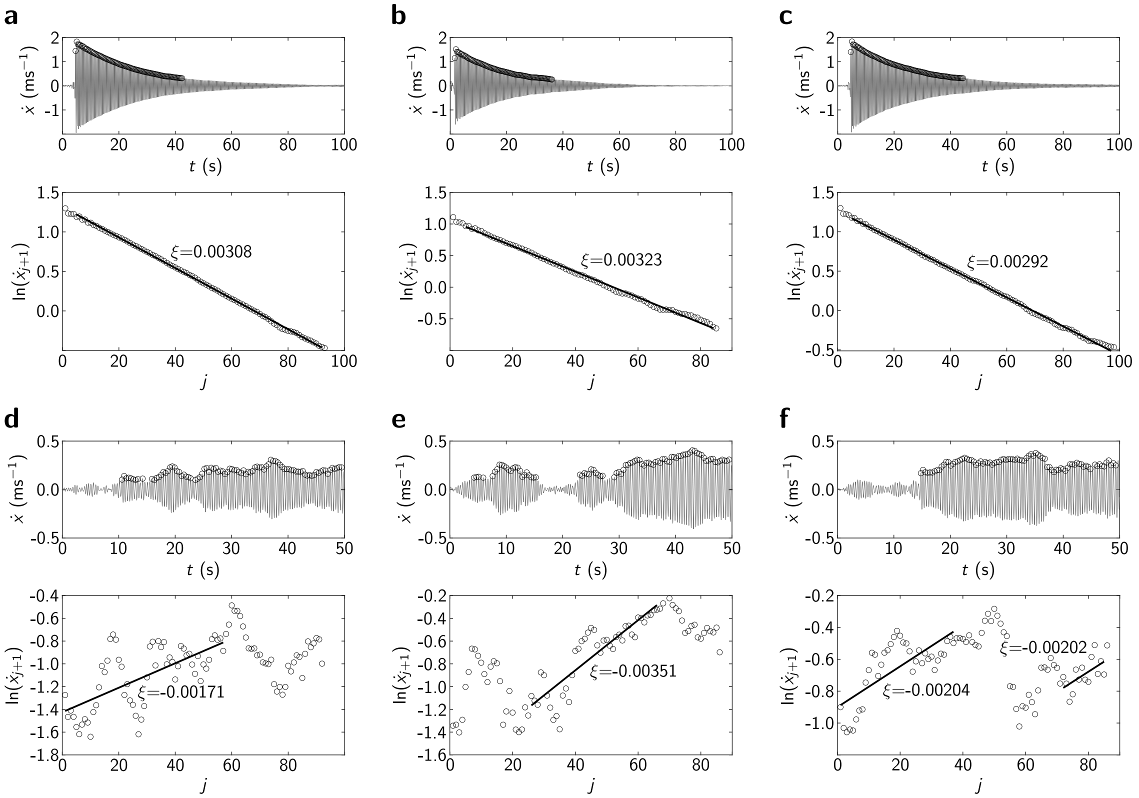

Supplementary Figure 6. **Complete data from the damping characterization experiments**. All subfigures feature a time trace, where peaks are highlighted by circular markers, and a logarithmic decrement plot, where the natural logarithm of the peak amplitude is plot against the peak number. (a–c) Free response experiments to measure the positive damping of the system. (d–f) Aerodynamic loading experiments to measure the negative damping of the system.

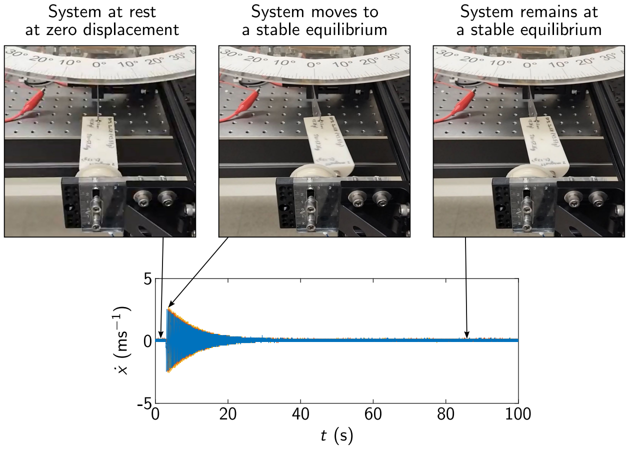

Supplementary Figure 7. **System's dynamic response as 24 V are provided to the coil, with the air flow blocked by the shutter**. The time history of the lateral velocity is accompanied by photos of the beam deformation.

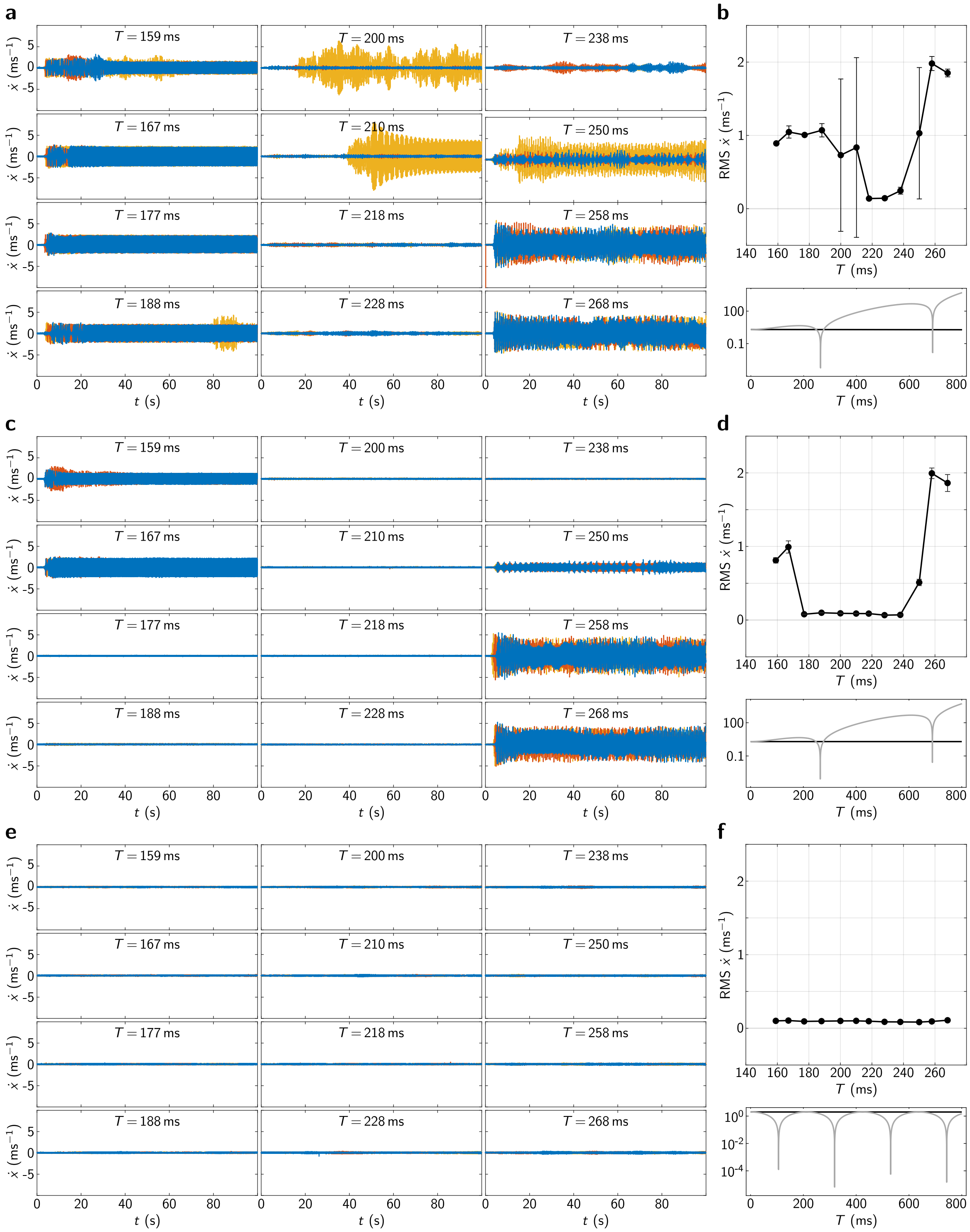

Supplementary Figure 8. **Additional results on switching dynamics**. Experimental time-histories, experimental RMS plots, and illustrations of the transcendental equation in condition (4b) that defines the emergence of antiresonance for the switched system (gray: $|\,\mathrm{tr}\Phi(T)\,|$ and black $1+\det\Phi(T)$), for the following switching scenarios: (a,b) $k_1 = -23.68$ N/m, $\xi_1 = 0.0031$, $k_2 = 10.74$ N/m, $\xi_2 = -0.0024$; (c,d) $k_1 = -23.68$ N/m, $\xi_1 = \xi_2 = 0.0031$, $k_2 = 10.74$ N/m; and (e,f) $k_1 = k_2 = 10.74$ N/m, $\xi_1 = 0.0031$, $\xi_2 = -0.0024$.

Supplementary Note 2. DESCRIPTIONS OF SUPPLEMENTARY VIDEOS

We provide videos for one of the trials of all the switching experiments reported in the main text and whose data is reported in Supplementary Figure 8a. Below, we report a list of the videos and a short description on what they show.

- Supplementary video 1 – Recording of the beam motion at switching period $T = 159$ ms.
- Supplementary video 2 – Recording of the beam motion at switching period $T = 167$ ms.
- Supplementary video 3 – Recording of the beam motion at switching period $T = 177$ ms.
- Supplementary video 4 – Recording of the beam motion at switching period $T = 188$ ms.
- Supplementary video 5 – Recording of the beam motion at switching period $T = 200$ ms.
- Supplementary video 6 – Recording of the beam motion at switching period $T = 210$ ms.
- Supplementary video 7 – Recording of the beam motion at switching period $T = 218$ ms.
- Supplementary video 8 – Recording of the beam motion at switching period $T = 228$ ms.
- Supplementary video 9 – Recording of the beam motion at switching period $T = 238$ ms.
- Supplementary video 10 – Recording of the beam motion at switching period $T = 250$ ms.
- Supplementary video 11 – Recording of the beam motion at switching period $T = 258$ ms.
- Supplementary video 12 – Recording of the beam motion at switching period $T = 268$ ms.

We also report a video documenting the unstable behavior of mode 1. Screenshots of this video are used in this SI, when documenting the system's instability in mode 1 (Supplementary Figure 7).

- Supplementary video 13 – Recording of the beam motion when the magnetic coil is provided with 24 V.

Supplementary Note 3. COMPLETE SWITCHING EXPERIMENTS DATA AND ADDITIONAL RESULTS

In Supplementary Figure 8, we report additional data and results on our switching experiments. We present experimental data and comprehensive RMS plots for three switching scenarios, including the one described in the main manuscript, and compare these results to theoretical predictions. These additional results are included to offer further confidence into the validity of our experimental approach.

- In Supplementary Figure 8a,b, we present results and additional data for the same case reported in the main text, where we switch between a saddle ($k_1 = -23.68$ N/m and $\xi_1 = 0.0031$) and an unstable focus ($k_2 = 10.74$ N/m and $\xi_2 = -0.0024$). Examining the time traces for switching periods of 200, 210, and 250 ms in Supplementary Figure 8a supports that these "borderline" scenarios are characterized by widely different responses, explaining the large standard deviations of Supplementary Figure 8b. We also report the RMS of experimental time traces as a function of the switching period (top panel of Supplementary Figure 8b), along with theoretical predictions for antiresonance windows visually identified by dips in $|\operatorname{tr}\Phi(T)|$. Theoretical predictions use the mean values of the measured stiffness and damping (bottom panel of Supplementary Figure 8b, with stability occurring when $|\operatorname{tr}\Phi(T)|$, in gray, is below $1+\det\Phi(T)$, in black).
- In Supplementary Figure 8c,d, we show results for a scenario where the air flow is blocked by the shutter, and switching amounts to turning the magnetic coil on and off. This is equivalent to switching between a saddle ($k_1 = -23.68$ N/m and $\xi_1 = 0.0031$) and a stable focus ($k_2 = 10.74$ N/m and $\xi_2 = 0.0031$), with an unstable average. Predictably, the system behaves similarly to the case in which we switch between unstable modes, with the presence of an antiresonance window, larger than in the previous case (top panel of Supplementary Figure 8d). It is worth noting that these experiments without air flow show less noisy time traces than the case with air flow, confirming the destabilizing nature of the aerodynamic forcing. Theory confirms experiments, anticipating the onset of compact antiresonance windows.
- In Supplementary Figure 8e,f, we present results for the case in which the magnetic coil is set to an open circuit configuration, while the fan and the shutter are turned on. The result is a scenario where the first mode is a stable focus ($k_1 = 10.74$ N/m, $\xi_1 = 0.0031$) and the second one is an unstable focus ($k_2 = 10.74$ N/m, $\xi_2 = -0.0024$), with a stable average. The result is a landscape where all switching periods yield stability, both experimentally and theoretically.

REFERENCES

[1] I. Wickramasinghe and J. M. Berg, Vibrational control of mathieu's equation, in *2013 IEEE/ASME International Conference on Advanced Intelligent Mechatronics* (IEEE, 2013) pp. 686–691.

[2] J. M. Berg and I. M. Wickramasinghe, Vibrational control without averaging, Automatica **58**, 72 (2015).
