# Supplementary material for: Dynamic stabilization of a mechanical oscillator in the absence of any stable feature
Source: Nat Commun. 2026 Mar 10;17:6024. doi: 10.1038/s41467-026-70493-1 (PMC13346612; doi:10.1038/s41467-026-70493-1)
Supplement: Supplementary file 2 — Description of Additional Supplementary Files [file 41467_2026_70493_MOESM2_ESM.pdf]

Description of Additional Supplementary Files

Supplementary Video 1: Recording of the beam motion at switching period T=159 ms.

Supplementary Video 2: Recording of the beam motion at switching period T=167 ms.

Supplementary Video 3: Recording of the beam motion at switching period T=177 ms.

Supplementary Video 4: Recording of the beam motion at switching period T=188 ms.

Supplementary Video 5: Recording of the beam motion at switching period T=200 ms.

Supplementary Video 6: Recording of the beam motion at switching period T=210 ms.

Supplementary Video 7: Recording of the beam motion at switching period T=218 ms.

Supplementary Video 8: Recording of the beam motion at switching period T=228 ms.

Supplementary Video 9: Recording of the beam motion at switching period T=238 ms.

Supplementary Video 10: Recording of the beam motion at switching period T=250 ms.

Supplementary Video 11: Recording of the beam motion at switching period T=258 ms.

Supplementary Video 12: Recording of the beam motion at switching period T=268 ms.

Supplementary Video 13: Recording of the beam motion when the magnetic coil is provided with 24 V.
